# Directed Evolution of a Highly Specific FN3 Monobody to the SH3 Domain of Human Lyn Tyrosine Kinase

**DOI:** 10.1371/journal.pone.0145872

**Published:** 2016-01-05

**Authors:** Renhua Huang, Pete Fang, Zengping Hao, Brian K. Kay

**Affiliations:** Department of Biological Sciences, University of Illinois at Chicago, Chicago, Illinois, United States of America; University of Rome Tor Vergata, ITALY

## Abstract

Affinity reagents of high affinity and specificity are very useful for studying the subcellular locations and quantities of individual proteins. To generate high-quality affinity reagents for human Lyn tyrosine kinase, a phage display library of fibronectin type III (FN3) monobodies was affinity selected with a recombinant form of the Lyn SH3 domain. While a highly specific monobody, TA8, was initially isolated, we chose to improve its affinity through directed evolution. A secondary library of 1.2 × 10^9^ variants was constructed and screened by affinity selection, yielding three variants, two of which have affinities of ~ 40 nM, a 130-fold increase over the original TA8 monobody. One of the variants, 2H7, displayed high specificity to the Lyn SH3 domain, as shown by ELISA and probing arrays of 150 SH3 domains. Furthermore, the 2H7 monobody was able to pull down endogenous Lyn from a lysate of Burkitt's lymphoma cells, thereby demonstrating its utility as an affinity reagent for detecting Lyn in a complex biological mixture.

## Introduction

**Fig 1:**
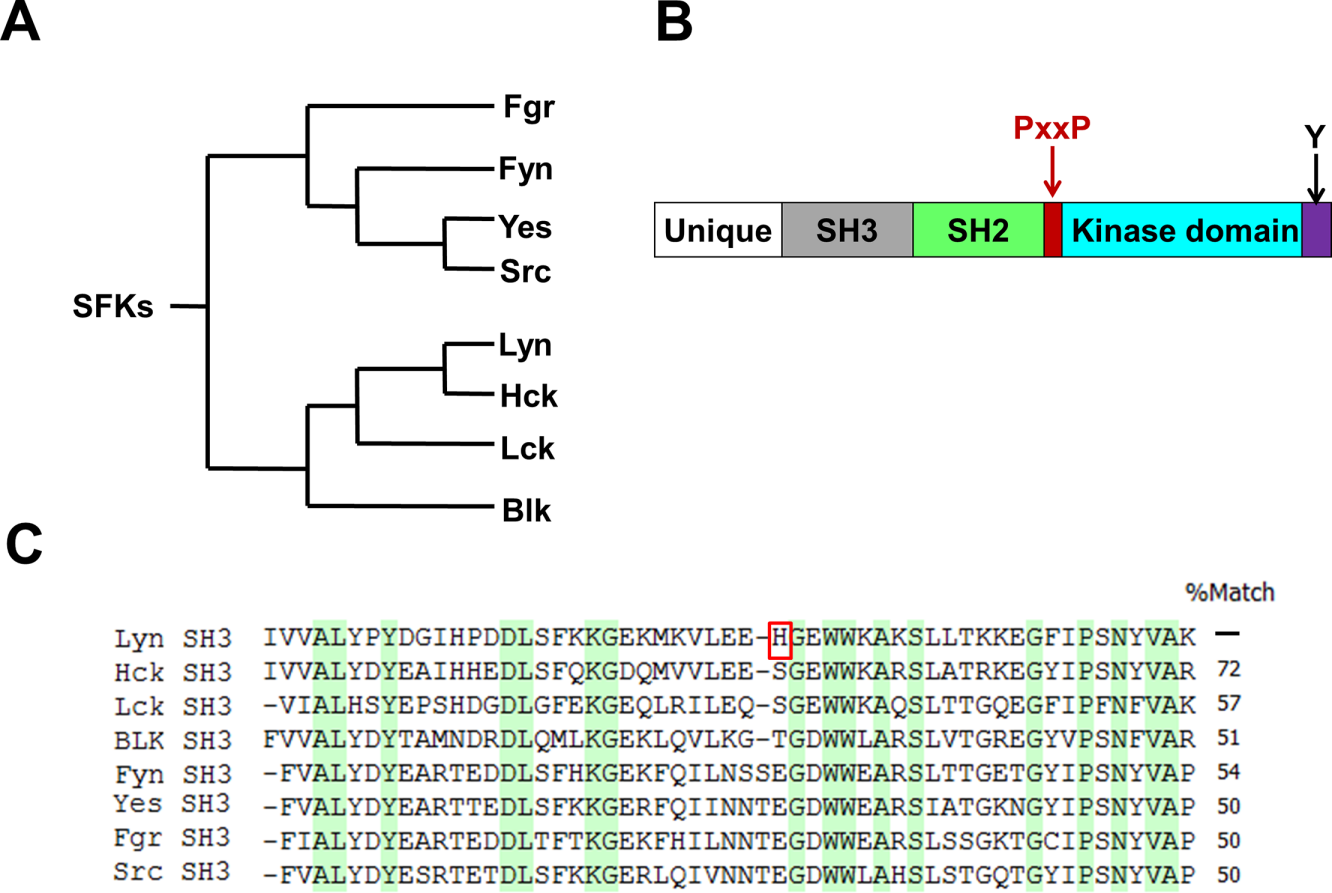
Evolutionary relatedness of Src family kinases (SFKs) and their common architecture. **(A)** A phylogenetic tree of human SFKs, based on the similarity of their kinase domains; adapted from another publication [4]. **(B)** The architecture of SFKs: an N-terminal unique region, a Src Homology 3 (SH3) domain, a Src Homology 2 (SH2) domain, a proline-containing linker region (PxxP), a kinase domain, and a C-terminal tyrosine (Y) residue. **(C)** The sequences of the SH3 domains of the human SFKs are aligned to reveal their amino acid identity relative to the Lyn SH3 domain; residues conserved among the SFKs members are highlighted in green columns and dashes are inserted to maximize the sequence alignment. The histidine residue (H30) that is unique to the n-Src loop of the Lyn SH3 domain is highlighted by a red square.

Src family kinases (SFKs) are active participants of many cell signaling pathways [1] and have been implicated in a wide variety of diseases, especially cancer [2]. The SFKs consist of 8 members, Blk, Fgr, Fyn, Hck, Lck, Lyn, Src, and Yes, in humans, and are likely the consequence of gene duplication [3]. Based on the sequence identity of the kinase domain [4], SFKs can be grouped into two subgroups (Fig 1A): the Src A group (i.e., Fgr, Fyn, Src, and Yes) and the Src B group (i.e., Blk, Hck, Lck, and Lyn). One member, Lyn, is expressed in hematopoietic cells [5], where it plays an important role in regulating the activation of mast [6] and B cells [7], apoptosis [8], and wound response [9]. Elevated expression and activity of Lyn have also been associated with several types of cancers [10,11,12] and autoimmune diseases [13].

Recombinant affinity reagents have great utility in ongoing cell biology research. They are commonly based on antibody fragments, such as human single-chain Fragments of variable regions (scFvs) [14], fragments of antigen binding (Fabs) [15], or single-domain antibody fragments [16]. Alternative scaffolds, such as affibodies [17], lipocalins [18], designed ankyrin repeat proteins (DARPins) [19], and fibronectin type III (FN3) monobodies [20], are growing in popularity due to their favorable biochemical characteristics [19,21]. Large libraries of engineered scaffolds can be screened effectively through bacterial display [22], phage display [23], mRNA display [24], ribosome display [25], and yeast display [26]. As sequenced reagents, they offer renewability and opportunities for engineering, unlike most monoclonal and all polyclonal antibodies [27]. The FN3 monobody is 94 amino acids in size and contains seven beta-strands, which fold into a structure resembling the variable domain of the immunoglobulin heavy chain [20]. The FN3 scaffold has been engineered for binding to a wide variety of targets, such as ubiquitin [28], estrogen receptor [29], lysozyme [30], streptavidin [31], human small ubiquitin-like modifier (SUMO) [32], phosphorylated IκBα peptide [33], Abl SH2 domain [34], and EphA2 [35].

Several recombinant affinity reagents of FN3 scaffold have been generated to members of the SFKs. Monobodies, which have been generated to the Src Homology 3 (SH3) domain of Src and Fyn, have been used as a biosensor of kinase activation [36] and as a reagent for biochemical assays [37], respectively. As no such recombinant reagent existed for Lyn, we set out to identify a tight and selective binder that could pull down the endogenous protein of human cells. Affinity selection of a phage library of FN3 monobodies led to the identification of several binders, one of which was improved 130-fold in affinity by mutagenesis, without loss of specificity. One affinity-matured monobody, 2H7, was used to pull down endogenous Lyn kinase from cultured Burkitt's lymphoma cells. Such a reagent offers utility in future applications such as biosensors and diagnostics.

## Materials and Methods

### Bacterial strains, plasmids, and phagemids

The BL21-DE3 (*fhuA2 [lon] ompT gal (λ DE3) [dcm] ΔhsdS*) and C41-DE3 (F–*ompT hsdSB (rB*^*-*^ *mB*^*-*^*) gal dcm* (DE3)) strains of *E*. *coli* were purchased from Novagen (Madison, WI) and Lucigen (Middleton, WI), respectively. The TG1 electrocompetent cells ([F' *traD36 proAB lacIqZ ΔM15] supE thi-1 Δ(lac-proAB) Δ(mcrB-hsdSM)5(rK*^*-*^ *mK*^-^*)*) from Lucigen (Middleton, WI) were used for construction of phage-display libraries. CJ236 bacterial cells (FΔ*(HindIII)*::*cat* (Tra^+^ Pil^+^ Cam^R^)/ *ung-1 relA1 dut-1 thi-1 spoT1 mcrA*) from New England BioLabs (Ipswich, MA), which lack functional dUTPase and uracil-N glycosylase, were used for generating single-stranded DNA (ssDNA) template that contained uracil inserted in the place of thymine residues. Such uracilated ssDNA was used for Kunkel mutagenesis [38,39]. Plasmids derived from pGEX-2T, pKP300 [40], and pET14b were used for overexpressing proteins fused to glutathione-S-transferase (GST), alkaline phosphatase [41], and yeast small ubiquitin-like modifier (SUMO), respectively. The FLAG epitope, DYKDDDDK, was fused to the N-terminus of the FN3 coding region in the pKP300 phagemid, thereby permitting detection of the phage-displayed FN3 domain with an anti-FLAG monoclonal antibody (Sigma-Aldrich; St. Louis, MO, # F1804). The phagemid pHEN4 [31,42] was used for constructing the primary phage-display library, and the phagemid pKP300 [43] was used for constructing the secondary library.

### Protein overexpression and purification

The procedures for overexpressing SUMO and GST fusion proteins of SH3 domains were described previously [43]. For expressing alkaline phosphatase fusion proteins of the FN3 monobodies, BL21-DE3 cells (Novagen) containing the expression constructs were used to inoculate 300 mL low phosphate medium [40] with 50 μg/mL carbenicillin, and incubated at 30°C at 270 rpm for 24 hours (h). Fusion proteins of SUMO and alkaline phosphatase were purified by immobilized metal affinity chromatography (IMAC) with nickel-nitriloacetic acid (Ni-NTA) resin (Qiagen; Valencia, CA, # 30250). For purification of the GST-SH3 proteins, clarified bacterial cell lysates were incubated with GST-bind resin (GE Healthcare Life Sciences; Piscataway, NJ, # 17-0756-01), and the captured fusion proteins were purified, as described elsewhere [44].

### Affinity selection of the primary FN3 library

The construction of the FN3 gene and the primary phage library was detailed in a previous study [31]. The GST-Lyn SH3 domain was chemically biotinylated [45] with the EZ-link NHS biotinylation kit (Thermo Fisher Scientific; Waltham, MA, # PI-21217) and used as a target for three rounds of affinity selection. All of the affinity selection steps were performed at room temperature, according to a published protocol [31]. After the third round of selection, bacterial single colonies were picked for phage amplification, followed by phage enzyme-linked immunosorbent assay (ELISA) to identify binders to the Lyn SH3 domain. Clones that yielded a positive ELISA signal were sequenced and further characterized by enzyme-linked binding assays (ELBA).

### ELBA and ELISA

Binding of virions and overexpressed monobodies was monitored in microtiter plate wells, as described previously [43]. An ELBA was performed, with published protocols [31,43], to evaluate the specificities of clones isolated from the primary library. This was accomplished through several steps, which are briefly described here. First, GST fusions to the SH3 domains of Lyn, Hck, Src, and Yes were diluted in phosphate buffered saline (PBS; 137 mM NaCl, 3 mM KCl, 8 mM Na_2_HPO_4_, 1.5 mM KH_2_PO_4_) to 5 μg/mL and distributed to triplicate wells of a Nunc Maxisorp microtiter plate (Thermo Fisher Scientific, # 44240421), for overnight incubation at 4°C. As controls, bovine serum albumin (BSA) and the anti-FLAG antibody (Sigma-Aldrich) were added to separate wells. The next day, the non-specific binding sites in the wells were blocked with casein (Thermo Fisher Scientific, # PI-37528) for 1 h, followed by addition of FN3-alkaline phosphatase fusion protein of TA1, TA7 and TA8 that had been diluted to 5 μg/mL in PBS containing 0.1% Tween 20. After 1 h incubation shaken at 200 rpm, the microtiter plate was washed five times with PBS + 0.1% Tween 20 and loaded with a chromogenic substrate, para-nitrophenyl phosphate (pNPP; Sigma-Aldrich, # N9389). The resulting absorbance was measured at 405 nm with a microtiter plate spectrophotometer (BMG Labtech; Cary, NC).

For assessing the specificities of three TA8 variants, 2H7, 2H10 and 3C12, phage ELISA was performed. First, a Nunc microtiter plate was first coated with SUMO-SH3 domains of Lyn, Hck and Btk at 5 μg/mL. The blocking reagent of casein served as the negative control. The ELISA assay was performed as described [43], and it was run in triplicate. Similar phage ELISA was performed for evaluating the specificity of 2H7 to the SH3 domains of all SFKs. In this assay, 1F11, a monobody that binds to several SFKs SH3 domains [31], was included to assess the quality of the immobilized SH3 domains. An anti His_6_-tag antibody conjugated to horseradish peroxidase (HRP) (Sigma-Aldrich, # A7058) was also included to normalize the amount of SH3 domain proteins immobilized in the microtiter plate wells.

### Comparing the binding site of monobodies relative to a peptide ligand

To determine if the isolated monobodies bound to the Lyn SH3 domain at the same location used to bind a proline-rich peptide [46], competition assays were devised. For TA8 monobody, an ELBA was performed with the purified fusion protein of the TA8-alkaline phosphatase. The GST-Lyn SH3 domain was immobilized on the surface of the Nunc plate wells by coating the wells with 5 μg/mL protein at 4°C overnight. After blocking, a proline-rich peptide, GMPT**P**PL**P**PRPANLGERQA, corresponding to a portion of the tyrosine kinase interacting protein (Tip) of herpesvirus saimiri, was added over a range of concentrations (0.1 μM to 40 μM), along with the TA8-alkaline phosphatase fusion to the wells. After 1 h incubation, the wells were washed with PBS + 0.1% Tween 20, and the TA8-alkaline phosphatase retained in the wells was detected with the chromogenic substrate, pNPP. The resulting absorbance was measured at 405 nm wavelength with a microtiter plate spectrophotometer. In parallel experiments, negative controls were a peptide (GMPT**A**PL**A**PRPANLGERQA) segment of Tip, in which two proline residues were substituted with alanine residues, and a third non-related peptide (DYKDDDDKLTVYHSKVNLP) of the same length. The signal at a particular competition point was divided by the signal of the wells without competing peptide, converted to percentage, and plotted against the molar concentration of the peptide used for this competition assay. For the 2H7 monobody, a similar competition experiment was performed except for two differences: first, it was a phage ELISA instead of an ELBA, and second, there were only two peptides used in the competition experiment (Tip and its control peptide) with the concentration range of the added peptides being 0.1 μM to 100 μM.

### Probing SH3 domain arrays

For probing the arrays with monobodies TA8 and 1F11, four polyvinylidene fluoride (PVDF) membranes, spotted with 150 human SH3 domains, were purchased from Panomics (Santa Clara, CA). The 150 human SH3 domains, overexpressed in *E*. *coli* as GST fusion proteins, were spotted in duplicate on four arrays, which were processed according to the manufacturer’s instructions, except that purified fusion proteins of FN3-alkaline phosphatase were used in lieu of primary and secondary reagents. After overnight incubation with 1 nM fusion protein at 4°C, the arrays were washed 10 times with PBS + 0.1% Tween 20, and then incubated with a substrate for enhanced chemifluorescence (GE Healthcare Life Sciences, # RPN2106). The four arrays were scanned simultaneously with a blue fluorescence filter on the Storm 860 PhosphorImager (GE Healthcare Life Sciences). The signal intensities of the spots were quantified with ImageQuant (GE Healthcare Life Sciences). Probing assay for the 2H7 monobody was similarly performed, except that the image was obtained with the Odyssey Fc imager (LI-COR Biosciences; Lincoln, NE). The signal intensities were quantitated with Image Studio Lite 4.0 (LI-COR Biosciences).

### Alanine scanning experiments

To determine the binding contribution of each amino acid residue in the variable loops of TA8, alanine scanning was performed [47], in which TA8 mutants with the alanine replacement were created with Kunkel mutagenesis [39]. Bacteria containing ten different TA8 mutants were inoculated into low phosphate media [40] for protein expression and purification with Ni-NTA resin (Qiagen). Purity of the purified protein samples was determined by sodium dodecyl sulfate -polyacrylamide gel electrophoresis (SDS-PAGE) and their concentrations were determined by the Bradford assay [48]. ELBA was performed as described above for the peptide competition experiment and data were normalized to the binding of the wild-type TA8 monobody. The anti-FLAG antibody was used to normalize the amount of FN3-alkaline phosphatase fusion proteins present in the microtiter plate wells.

### Affinity maturation of TA8 monobody

A mutagenic library of TA8 monobody was constructed by Kunkel mutagenesis [39]. Briefly, a pKP300 phagemid [40] carrying the FN3 gene fused to the N-terminus of a truncated gene III of M13 bacteriophage was transformed into CJ236 cells for isolating uracilated single-stranded DNA (ssDNA). Two mutagenic oligonucleotides (IDT DNA; Coralville, IA), containing NNK codons for 6 selected residues (three per loop), were phosphorylated and annealed to the ssDNA template, which was converted to double-stranded DNA (dsDNA) *in vitro*. The resulting dsDNA was introduced into TG1 competent cells (Lucigen) by electroporation, and after recovery for 30 min at 37°C and 200 rpm, the recovered cells were spread on the 2 × YT agar plate with 50 μg/mL carbenicillin for overnight incubation at 30°C. The next day, to estimate the diversity of the library, the number of total transformants was calculated and sixteen transformants were randomly picked for sequencing to determine recombination rate.

The bacterial mutant library of TA8 was amplified as described elsewhere [39]. Virions from overnight culture were pelleted with polyethylene glycol (PEG) 8000, and resuspended in Tris-buffered saline (TBS; 50 mM Tris-HCl, 150 mM NaCl, pH 7.5) containing 0.5% Tween 20 (volume/volume) and 0.5% bovine serum albumin (BSA; mass/volume). The virions were then mixed with the biotinylated Lyn SH3 domain (300 nM, final concentration) and the non-biotinylated SH3 domain of Hck and Bruton's tyrosine kinase (Btk) (both at 600 nM final concentration). After 1 h incubation, streptavidin-coated magnetic beads (Promega; Madison, WI, # Z5482) were added to capture virion/protein complex. Beads were collected with a magnet, washed 7 times with PBS + 0.5% Tween 20, and 7 times with PBS + 0.1% Tween 20 that was supplemented with non-biotinylated proteins of 300 nM Lyn SH3 domain, 10 μM Btk SH3 domain and 2.4 μM of Hck SH3 domain. The steps of recovering bound virions, infecting TG1 cells, collecting infected cells, and amplifying the virions were performed as described previously [43]. The second round of affinity selection was carried out in the same manner, except the concentration of the biotinylated target protein of the Lyn SH3 domain was reduced to 3 nM.

### Isothermal titration calorimetry (ITC)

SUMO fusion proteins of FN3 monobodies and the Lyn SH3 domain were purified to homogeneity of > 95% and dialyzed against 4 liters (L) of 25 mM Tris-HCl (pH 7.5), 150 mM NaCl, and 100 mM imidazole. After the first round of 2 h dialysis, the protein samples were transferred to another 4 L of fresh dialysis buffer, and after another 2 h, they were transferred again to 4 L of fresh buffer for overnight dialysis. The next morning, the protein concentrations of dialyzed samples were measured with a NanoDrop ND-1000 spectrophotometer (Thermo Fisher Scientific). The protein samples were then degassed by bench top centrifugation at 16,000 × g for 1 min and added to the sample cell (200 μL) and syringe (40 μL) of an ITC200 instrument (GE Healthcare Life Sciences). The Lyn SH3 domain was loaded into the sample cell at 50 μM and FN3 monobodies were loaded into the syringe at 500 μM. The reference cell was loaded with water. FN3 monobodies were injected into the cell at 2 μL per injection at 25°C, with a reference power of 10 μcal/s. The heat change of each injection was recorded and analyzed with Origin software (GE Healthcare Life Sciences).

### Cell culturing and pull-down experiment

The Ramos cell line of Burkitt's lymphoma cells was purchased (ATCC; Manassas, VA, # CRL-1596), and propagated according to the distributer’s instructions. The cells were passaged in culture plates every two days, and after three passages, ~ 4 x 10^5^ cells were harvested. Collected cells were lysed in 200 μL of RIPA buffer (25 mM Tris-HCl of pH 7.6, 150 mM NaCl, 1% NP-40, 1% sodium deoxycholate, and 0.1% sodium deoxycholate sulfate), which had been supplemented with 1% Triton and protease inhibitors (Roche Life Sciences; Indianapolis, IN, # 11873580001). After incubation on ice for 2 h, the lysate was clarified by centrifugation at 16,000 × g for 10 min. The final supernatant was divided into two equal aliquots (i.e., 100 μL) and each was mixed with 1.5 nanomole of biotinylated form of either the 2H7 or the wild-type FN3 (WT-FN3) monobody and incubated overnight at 4°C. The next morning, streptavidin-coated magnetic beads (Promega) were washed three times with PBS, blocked for 30 min with casein (Thermo Fisher Scientific), and added to the mixtures of the biotinylated monobodies and cell lysate. After 30 min tumbling at room temperature, the magnetic beads were collected in a magnetic stand and washed four times with the RIPA + 1% Triton. The washed beads were resuspended in 30 μL of the SDS loading buffer and boiled at 95°C for 10 min. The boiled samples, separated from the magnetic beads, and 170 femtomole of recombinant Lyn kinase (Invitrogen; Carlsbad, California, # P2907) were loaded to SDS-PAGE gel for electrophoresis. The resolved proteins were transferred to the nitrocellulose membrane for Western blotting. After the transfer, the nitrocellulose membrane was rinsed once with distilled water and blocked for 1 h with a blocking buffer (LI-COR Biosciences, # 927–40000), followed by addition of an anti-Lyn mouse monoclonal antibody (Santa Cruz Biotech; Santa Cruz, CA, # sc-376100) for 1 h incubation. After several washes, the blot was incubated with a goat anti-Mouse IgG conjugated to infrared dye IRDye 800 (LI-COR Biosciences, # 925–32210). Then blot was washed three times with PBS + 0.1% tween and scanned with the Odyssey Fc imager (LI-COR Biosciences).

### Kinase assay

The kinase assay was performed with a Beacon Tyrosine kinase assay kit (Invitrogen, A-35725) according to the manufacturer’s instructions. The kinase buffer was prepared by supplementing the provided buffer with dithiothreitol (DTT) (Final concentration: 2 mM) and dimethyl sulfoxide (DMSO) (Final concentration: 0.1%). In the kinase buffer, Dasatinib (Final concentration: 10 μM; Selleck Chemicals; Houston, TX, # S1021), 2H7 and wild-type FN3 (WT-FN3) (Final concentration for both: 7.5 μM) were mixed with Lyn kinase (final concentration: 105 nM; Invitrogen, # P2907). The mixture was incubated at 4°C for 30 min and then at room temperature for 20 min. The detection complex was prepared by mixing the Oregon green, an anti-phosphopeptide antibody and a peptide substrate. As a positive control (highest fluorescence signal), the anti-phosphopeptide antibody was excluded in the reaction, as a negative control (lowest fluorescence signal), the recombinant Lyn kinase was excluded in the reaction. The detection complex was added to the mixtures prepared above, followed by 10 min incubation at 30°C and then the addition of ATP. After another 12 min incubation at 30°C, each reaction was loaded into three adjacent wells of a non-binding microtiter plate (Corning; New York, NY, # 3915) and scanned by POLARstar OPTIMA plate reader (BMG LABTECH).

## Results

### Conserved structure of SFKs and sequence alignment of their SH3 domains

SFKs is a conserved family of tyrosine kinases (Fig 1A) and its members are highly similar in overall structure, which consists of an N-terminal unique domain, a SH3 domain, a SH2 domain, a proline-rich linker, a kinase domain and a C-terminal tail that contains a conserved tyrosine residue (Fig 1B). Each individual domain is highly conserved among the family; for example, the SFKs SH3 domains share 50% to 72% amino acid identity with the Lyn SH3 domain (Fig 1C). This degree of conservation makes it challenging to generate a recombinant affinity reagent that is specific for Lyn.

### Isolation of monobodies binding to the Lyn SH3 domain

**Fig 2:**
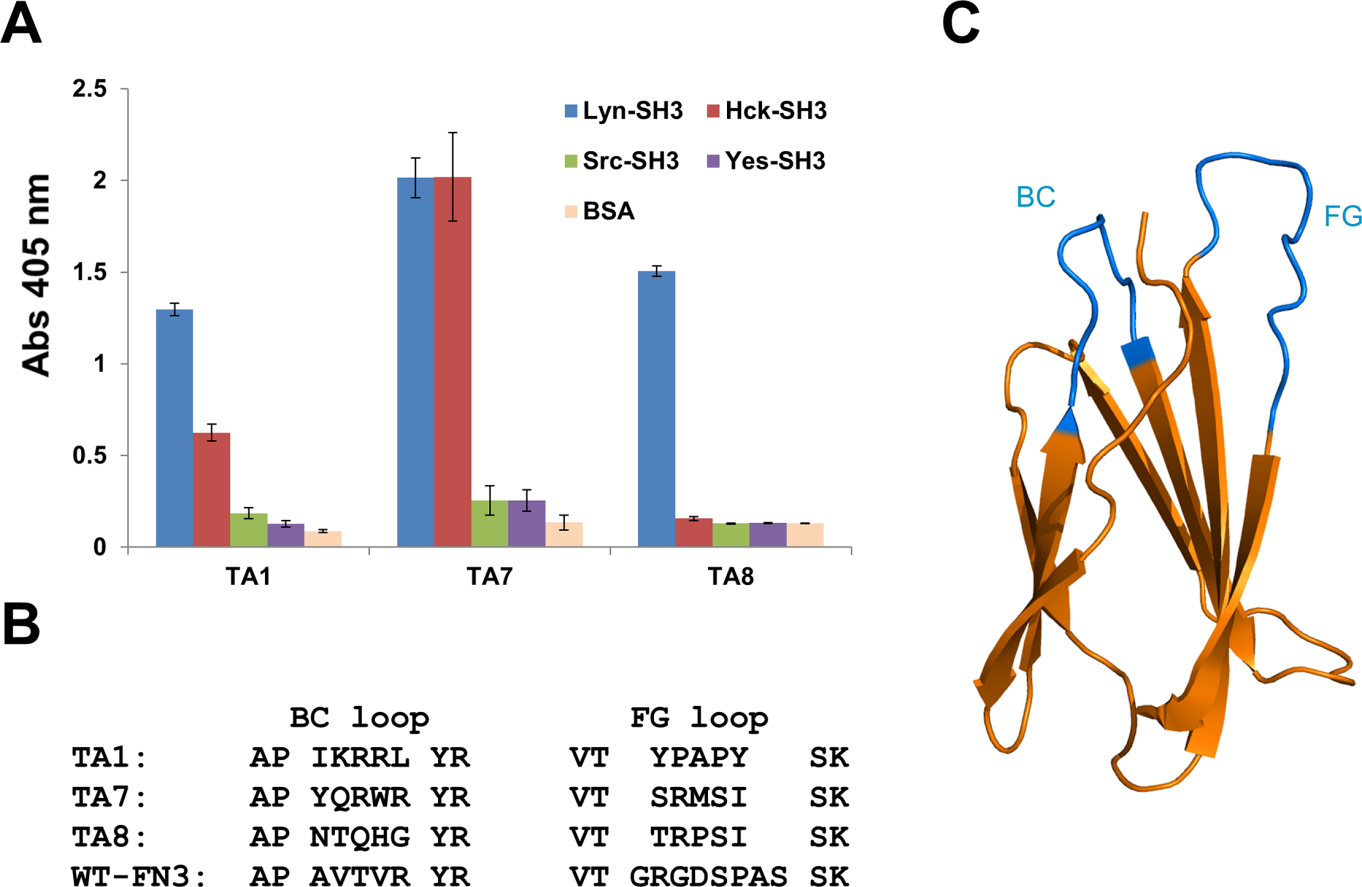
Enzyme-linked binding assay (ELBA) for testing specificities of three FN3 monobodies and sequence alignment of their variable loops. **(A)** The specificities of the TA1, TA7, and TA8 monobodies were evaluated by ELBA against four different SH3 domains of the SFKs. Bovine serum albumin (BSA) served as the negative target control. Absorbance values (Abs 405 nm) were normalized to the amount of the monobodies present in the wells with an anti-FLAG antibody. **(B)** The amino acid sequences of the variable loops of the three monobodies and wild-type FN3 (WT-FN3). **(C)** The three-dimensional cartoon of FN3 (PDB: 1TTG), as represented with the PyMol program, which is available from http://www.schrodinger.com/. The BC and FG variable loops are denoted in blue.

A primary phage library containing 2 × 10^9^ FN3 variants [31] was screened for clones binding to the SH3 domain of Lyn kinase. Three rounds of affinity selection were performed, yielding three monobodies, TA1, TA7 and TA8. These three monobodies were prepared in soluble form and examined in an enzyme-linked binding assay (ELBA), which revealed that while all three bound the Lyn SH3 domain well, TA1 and TA7 cross-reacted with Hck SH3 domain (Fig 2A), and TA8 bound selectively to the Lyn SH3 domain, but not to the SH3 domains of Hck, Src and Yes kinases (Fig 2A). Interestingly, unlike the canonical proline-rich motif (PxxP) present in many SH3 domain-binding peptides [49,50,51], all three monobodies lack PxxP motifs (Fig 2B) in their variable BC and FG loops (highlighted in blue of Fig 2C). Based on the initial demonstration of its specificity for the Lyn SH3 domain, monobody TA8 was selected for further characterization.

### Characterization of the TA8 monobody

**Fig 3:**
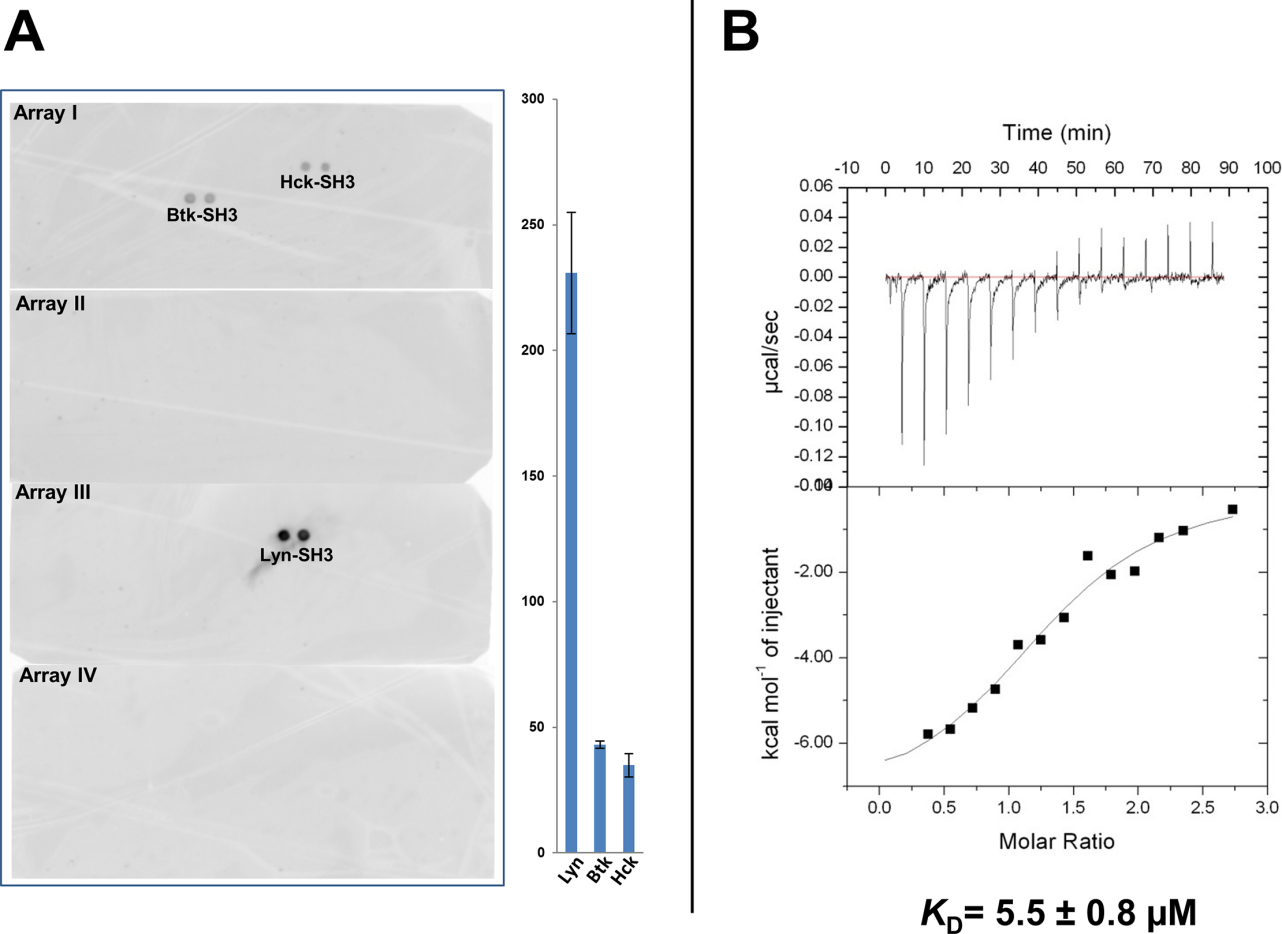
Characterization of the specificity and affinity of TA8 monobody. **(A)** Evaluating the specificity of TA8 by probing 150 human SH3 domains. Four arrays (Panomics) were probed with TA8 monobody and scanned simultaneously with a Storm860 imager (GE Healthcare Life Sciences). The signal intensities of the spots were quantified with the ImageQuant (GE Healthcare Life Sciences). Units in the histograms represent relative comparisons, with error bars corresponding to the standard deviations of the signal intensities of duplicate spots. **(B)** Measurement of the dissociation constant (*K*_D_) of TA8 binding to the Lyn SH3 domain by isothermal titration calorimetry (ITC). The thermogram (top panel) and the plotted titration curve (bottom panel) were acquired with an ITC200 (GE Healthcare Life Sciences). A representative experiment is shown here and the observed N, ΔH, and ΔS values are 1.32, -7.6 x 10^3^ cal/mol, and -1.38 cal/mol/deg, respectively. The ITC experiment was performed five times to generate an average *K*_D_ value of 5.5 ± 0.8 μM.

As there are 296 SH3 domains in the human proteome [52], it was desirable to test the specificity of the TA8 monobody against a much larger number of SH3 domains than we originally tested. To that purpose, we probed TA8 against commercially available arrays of 150 human SH3 domains. TA8 demonstrated an amazing degree of specificity, as it bound principally to the Lyn SH3 domain, and exhibited minimal cross-reactivity to the SH3 domains of Hck and Btk (Fig 3A). To demonstrate that other SH3 domains on the arrays were still functional, a pan-specific monobody, 1F11 [31], was used to probe the arrays and it revealed that 1F11 reacted with 36 different SH3 domains, including 7 of the 8 SFKs (S1 Fig).

While TA8 was shown to be highly selective, it must possess sufficient affinity (e.g., 60–250 nM) for it to be useful in pull-down experiments [31,53]. To determine if TA8 monobody met this requirement, it was overexpressed, purified, and used in isothermal titration calorimetry (ITC) experiments [54]. Five measurements yielded an average dissociation constant (*K*_D_) of 5.5 ± 0.8 μM; a typical ITC result is shown in Fig 3B. This value is similar to those of peptide ligands for binding to SH3 domains [49]. As this value was considered insufficient for pull-down experiments, affinity maturation experiments were deemed necessary to improve its affinity.

### Design and construction of a secondary library

**Fig 4:**
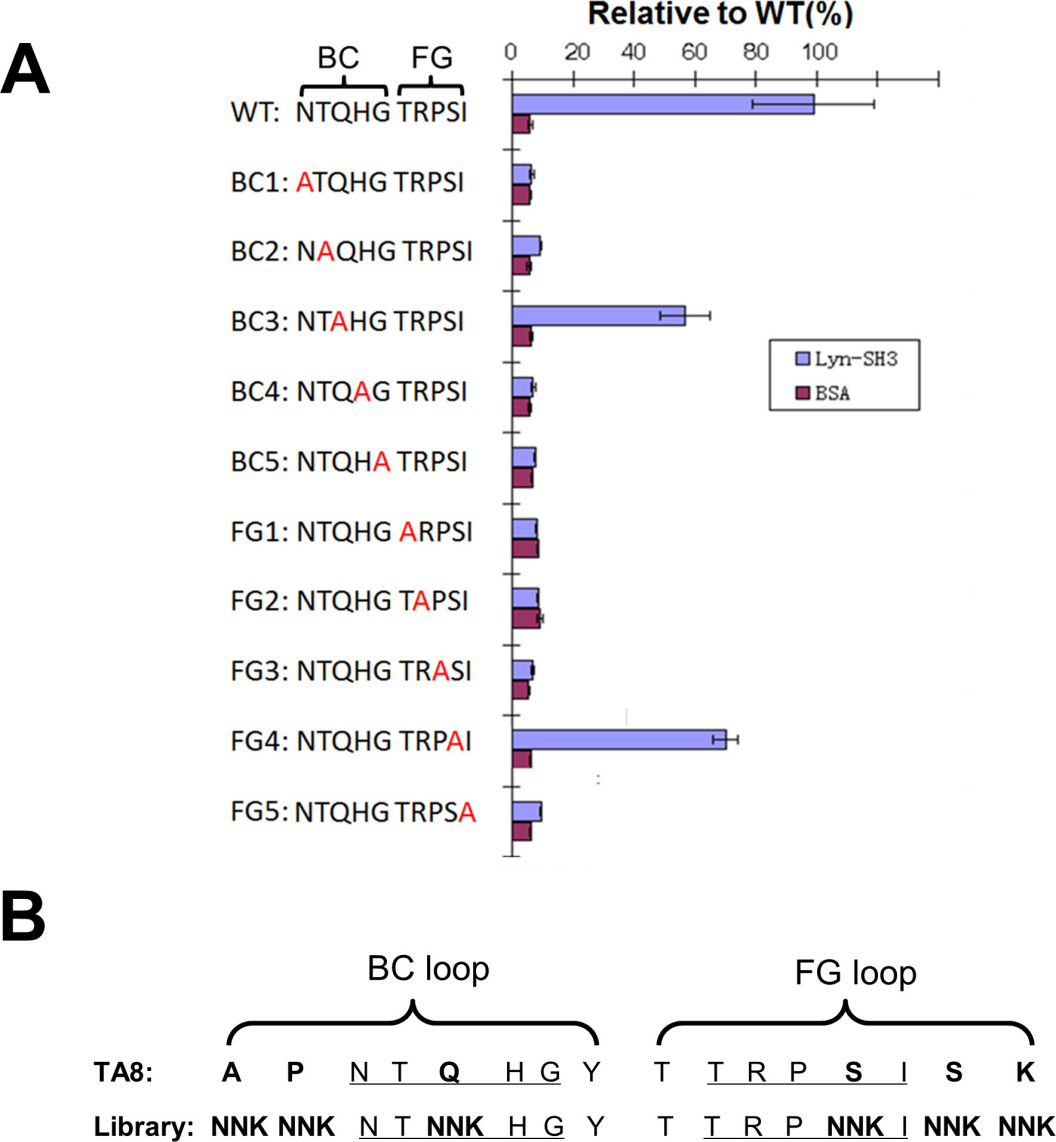
Alanine scanning and design of the secondary library. **(A)** Ten residues in the BC and FG loops of the wild-type (WT) TA8 clone were individually substituted with the alanine residue, and the binding of each variant to the Lyn SH3 domain was assessed by ELBA. Observed binding values were normalized to that of WT TA8 clone, with error bars corresponding to the standard deviations of triplicate measurements. BSA served as the negative control. **(B)** Six positions in the TA8 were randomized with NNK codons for constructing the secondary library. The underlined residues of each loop are the positions that were diversified in the primary library, from which the TA8 was isolated.

As the TA8 monobody exhibited an impressive specificity towards the Lyn SH3 domain, we decided to randomize only residues in the variable loops that were not crucial to binding. To identify such positions, alanine scanning was performed [47]. Two residues were demonstrated to be not crucial for binding: when either Gln in BC loop or Ser in FG loop was replaced with alanine, the variants retained 56% and 77% binding of the wild-type clone (Fig 4A), respectively, and, therefore, these two residues were chosen for randomization. To increase the sequence diversity of the secondary library, another four flanking residues (Ala and Pro of BC loop and Ser and Lys of FG loop), which had not been diversified in the original TA8 clone, were also randomized (Fig 4B).

A secondary library was constructed through Kunkel mutagenesis [38,39]. Sixteen clones of the 2.2 × 10^9^ transformants were selected for sequence analysis: 9 clones had mutations in both the BC and FG loops, suggesting that our secondary library had a diversity of 1.2 × 10^9^ members (2.2 × 10^9^ × 56%), which was large enough to cover most of the codon permutations (i.e., 32^6^ = 1.1 × 10^9^) anticipated for the six randomized positions.

### Affinity selection of the secondary library and characterization of output

**Fig 5:**
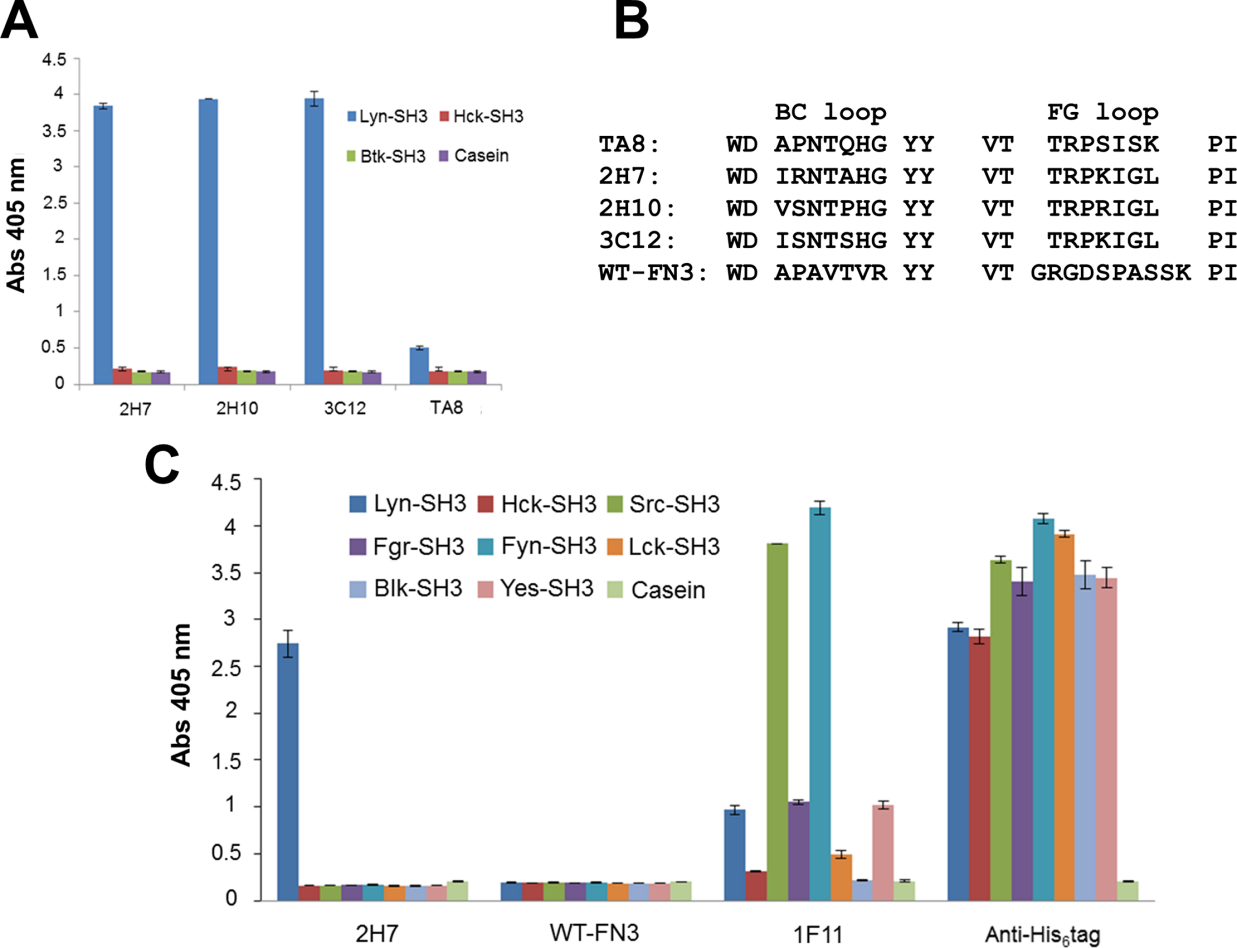
Phage ELISA for testing the specificities of TA8-derived variants and the alignment of their variable loop sequences. **(A)** ELISA for testing the specificities of TA8 and its three variants against the SH3 domains of Btk, Hck, and Lyn. Absorbance (405 nm) values were normalized to the display level of the FLAG-epitope on virions, with error bars corresponding to the standard deviations of triplicate measurements. Casein was the negative target control. **(B)** Amino acid sequences of the BC and FG loops of TA8, three variants, and wild-type FN3 (WT-FN3). **(C)** Phage ELISA for examining the specificity of one TA8 variant, 2H7. Microtiter plate wells were coated with equal amounts of SH3 domains (His_6_-tagged), as demonstrated by comparable binding of an anti-His_6_ tag antibody to the wells. Virions displaying WT-FN3 served as a negative control for phage-display. The binding of 1F11, a monobody that binds to several SFKs SH3 domains [31], was used as the internal control to monitor the functions of the immobilized SH3 domains. Error bars are the standard deviations of triplicate measurements.

Affinity selection of the secondary library with the Lyn SH3 domain was performed in the presence of excess soluble forms of Hck and Btk SH3 domains, which previously exhibited some cross-reactivity with the TA8 monobody. After two rounds of affinity selection, 184 clones were selected for phage ELISA, of which 65% had three-fold higher values to the Lyn SH3 domain than the original TA8. The best eight clones were selected for further evaluation, of which three (i.e., 2H7, 2H10 and 3C12) bound with the highest signals, while retaining their specificity for the Lyn SH3 domain (Fig 5A). Although all three variants have different sequences in their loops (Fig 5B), one position (TRP**S**ISK) in the FG loop seemed to favor arginine or lysine (K/R), whereas at another position (TRPSI**S**K) glycine appeared preferred.

**Fig 6:**
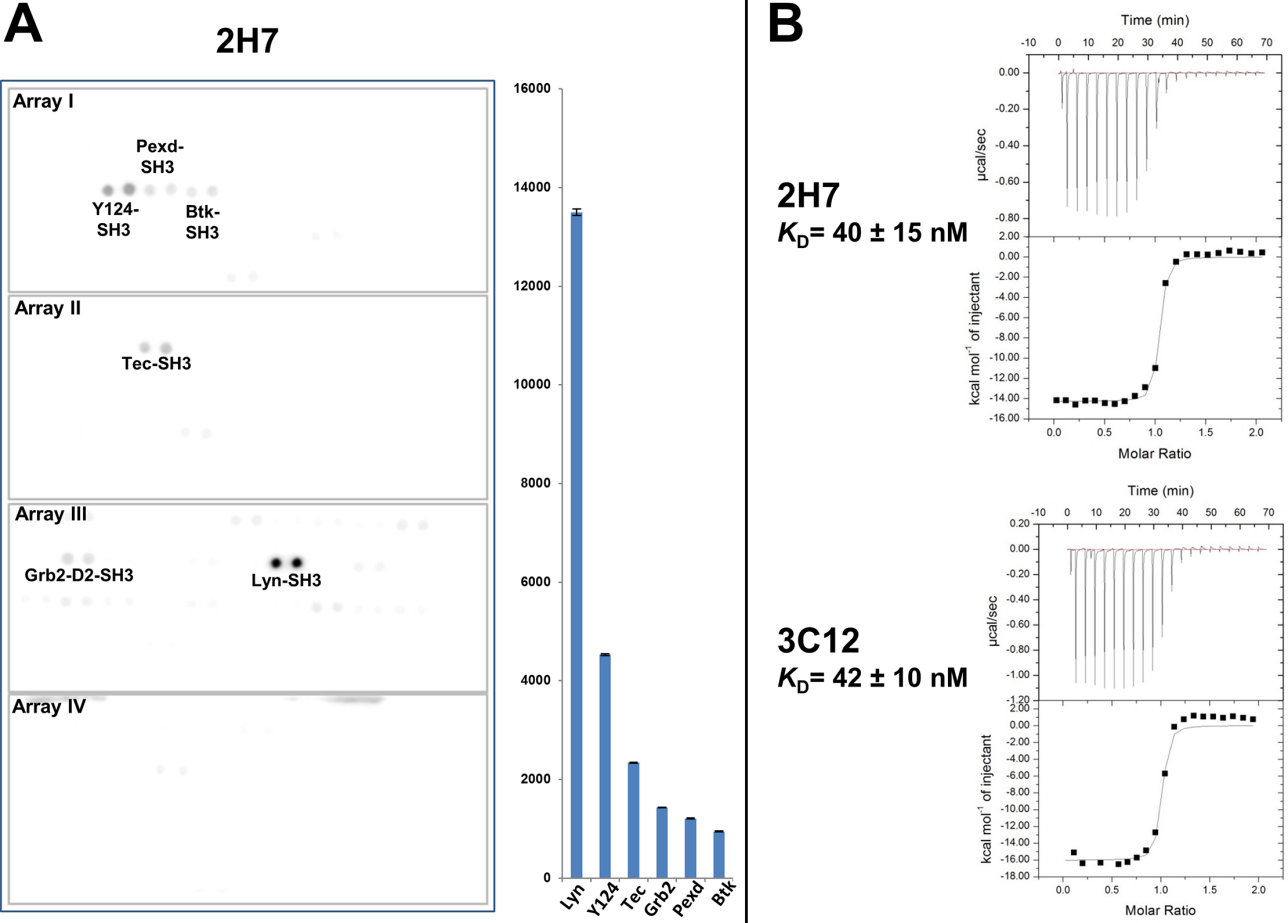
Characterization of two TA8-derived variants, 2H7 and 3C12. **(A)** Evaluating the specificity of the 2H7 monobody by probing arrays of 150 human SH3 domains, which have been spotted in duplicate. Values plotted on the y-axis of the histogram represent relative units, with error bars corresponding to the standard deviations of signal intensities of duplicate spots. **(B)** The dissociation constants (*K*_D_s) of two TA8 variants, 2H7 and 3C12, were determined by ITC. For the two representative experiments shown here, the observed N, ΔH, and ΔS values for 2H7 and 3C12 are 0.998, -1.43 × 10^4^ cal/mol, -13.3 cal/mol/deg and 0.966, -1.6 × 10^4^ cal/mol, -20.2 cal/mol/deg, respectively. The ITC experiments were performed three times for each variant; the average *K*_D_ values for 2H7 and 3C12 were measured to be 40 ± 15 and 42 ± 10 nM, respectively.

Clone 2H7 was picked for further evaluation. In phage ELISA, 2H7 bound only to the SH3 domain of Lyn out of all eight SFKs (Fig 5C). In contrast, the wild-type FN3 (WT-FN3) monobody did not bind to any SH3 domain, and the pan-specific monobody, 1F11 [31], bound to the SH3 domains of seven members of the SFKs. The above results suggest that 2H7 monobody specifically reacts with the Lyn SH3 domain, but not with any other SH3 domains of the SFKs. To evaluate the specificity of 2H7 clone further, the 2H7 protein was used to probe arrays of 150 human SH3 domains. The relative quantitation of binding showed that 2H7 bound to the Lyn SH3 domain at least 3-fold stronger than to any of the other 149 SH3 domains examined. Besides reacting with Lyn SH3 domain, 2H7 exhibited some cross-reactivity to the SH3 domains of Y124, Tec, Grb2-D2, PEXD, and Btk (listed in the order of signal intensity from high to low) as seen in Fig 6A.

To determine the *K*_D_ values of the 2H7 and 3C12 to the Lyn SH3 domain, ITC was performed. The *K*_D_ values of 2H7 and 3C12 for the Lyn SH3 domain were 40 ± 15 nM and 42 ± 10 nM (Fig 6B), respectively. The observations that 2H7 and 3C12 had similar *K*_D_ values and both had the same mutations in their FG loops suggest that the mutations in the FG loops, but not those in BC loops, are the main contributor to the increase in their affinities.

### Comparing the binding site of monobodies relative to a peptide ligand

**Fig 7:**
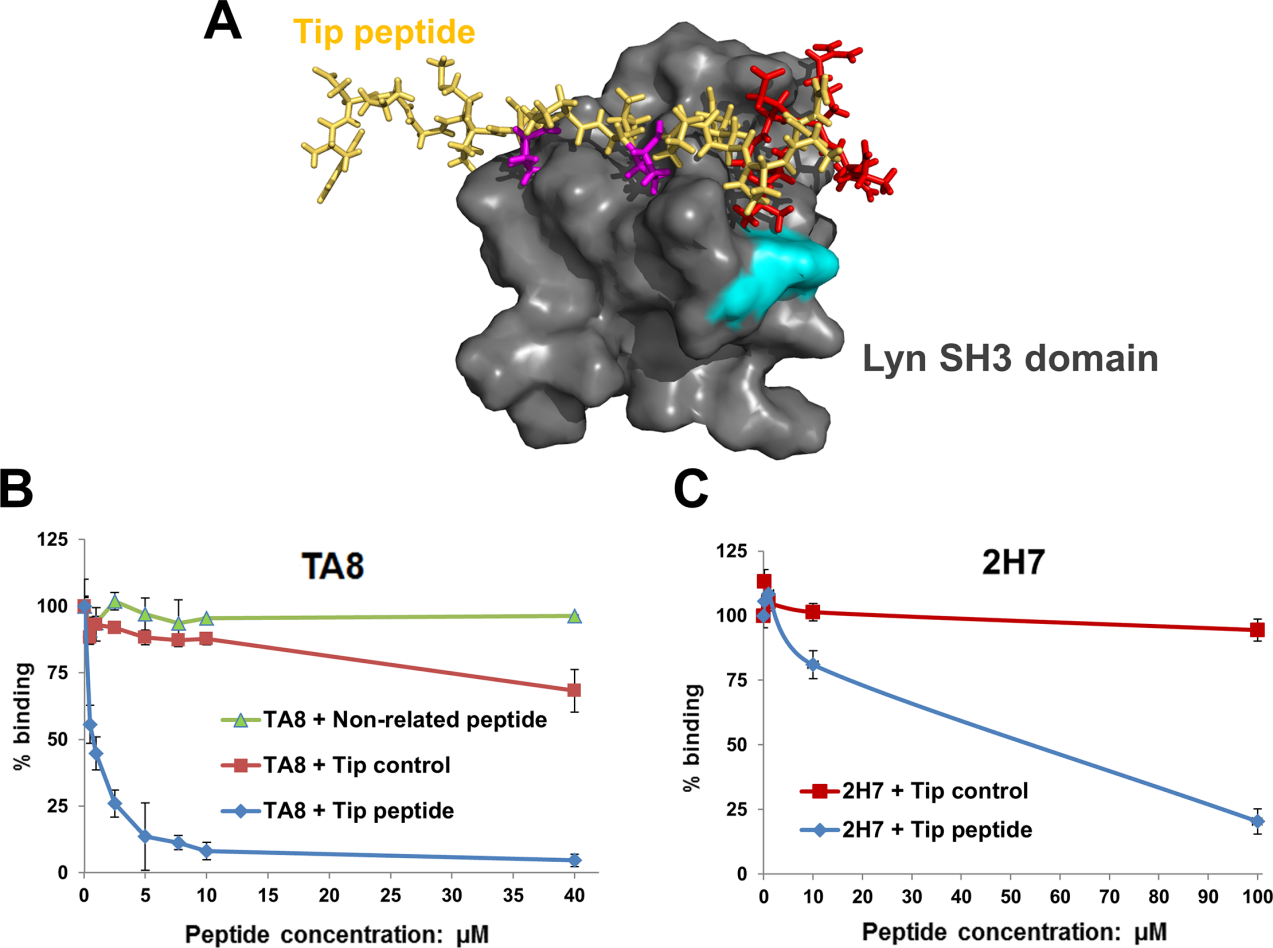
Mapping the binding location of two monobodies in comparison to a peptide ligand. **(A)** The NMR structure of the Lyn SH3 domain in complex with a peptide segment of the tyrosine kinase interacting protein (Tip) of herpesvirus saimiri (PDB: 1WA7). The peptide and SH3 domain are shown as a stick structure and a surface structure, respectively. In the Tip peptide (GMPT**P**PL**P**PRPAN**LGERQA**), two underlined proline residues are shown in purple and the underlined C-terminal sequence is shown in red. In the surface structure of Lyn SH3 domain, the Histidine-30 residue is highlighted in cyan. **(B)** Competition ELBA between the Tip peptide and TA8 monobody for binding to the Lyn SH3 domain. Two control peptides were used in the assay, the Tip control and a non-related peptide of the same length. The sequence of Tip control peptide is GMPT**A**PL**A**PRPANLGERQA, in which the two proline residues were both replaced with a alanine residue (underlined). The non-related peptide is another 19-mer peptide with an unrelated sequence (DYKDDDDKLTVYHSKVNLP). **(C)** Competition ELISA between the Tip peptide and 2H7 monobody for binding to the Lyn SH3 domain.

Many SH3 domains, including the Lyn SH3 domain, interact with proline-rich (PxxP) peptide regions within other proteins [46,51,55,56]. The SH3 domains bind to the PxxP sequences via a shallow groove that contains two binding pockets, one for each conserved proline residue [57,58]. As determined by nuclear magnetic resonance (NMR), the Lyn SH3 domain (Fig 7A) contains a similar groove that interacts with a class-II PxxP peptide (GMPT**P**PL**P**PRPANLGERQA) within the tyrosine kinase interacting protein (Tip) of herpesvirus saimiri [46]. To determine if TA8 and its variant, 2H7, bound the same location on the Lyn SH3 domain as the Tip peptide, competition assays were performed. The Tip peptide competed with both TA8 and 2H7 for binding to the Lyn SH3 domain (Fig 7B and 7C) in a concentration-dependent manner, implying that both TA8 and 2H7 bind to the same or overlapping region on the Lyn SH3 domain as the Tip peptide does.

The NMR derived three-dimensional structure of Tip peptide with Lyn SH3 domain shows that the C-terminal sequence of Tip peptide (GMPTPPLPPRPAN**LGERQA**, highlighted in red in Fig 7A) interacts with a groove (a region close to the cyan-highlighted H30 in Fig 7A) of the Lyn SH3 domain that is outside the canonical proline-binding pockets. This interaction contributed to the higher specificity of Tip peptide towards the Lyn SH3 domain among the closely related members of SKFs [59]. In the competition ELBA of TA8, the Tip control peptide (GMPT**A**PL**A**PRPAN**LGERQA**), which still has the same C-terminal sequence of the Tip peptide, moderately competed with TA8 for binding to the Lyn SH3 domain (Fig 7B). This observation suggests that the TA8 may also interact with this non-canonical region [60] on the Lyn SH3 domain and this interaction may also account for how TA8 achieves its specificity to the Lyn SH3 domain among SFKs, as the Tip peptide does.

### Pull-down experiment and kinase assay

**Fig 8:**
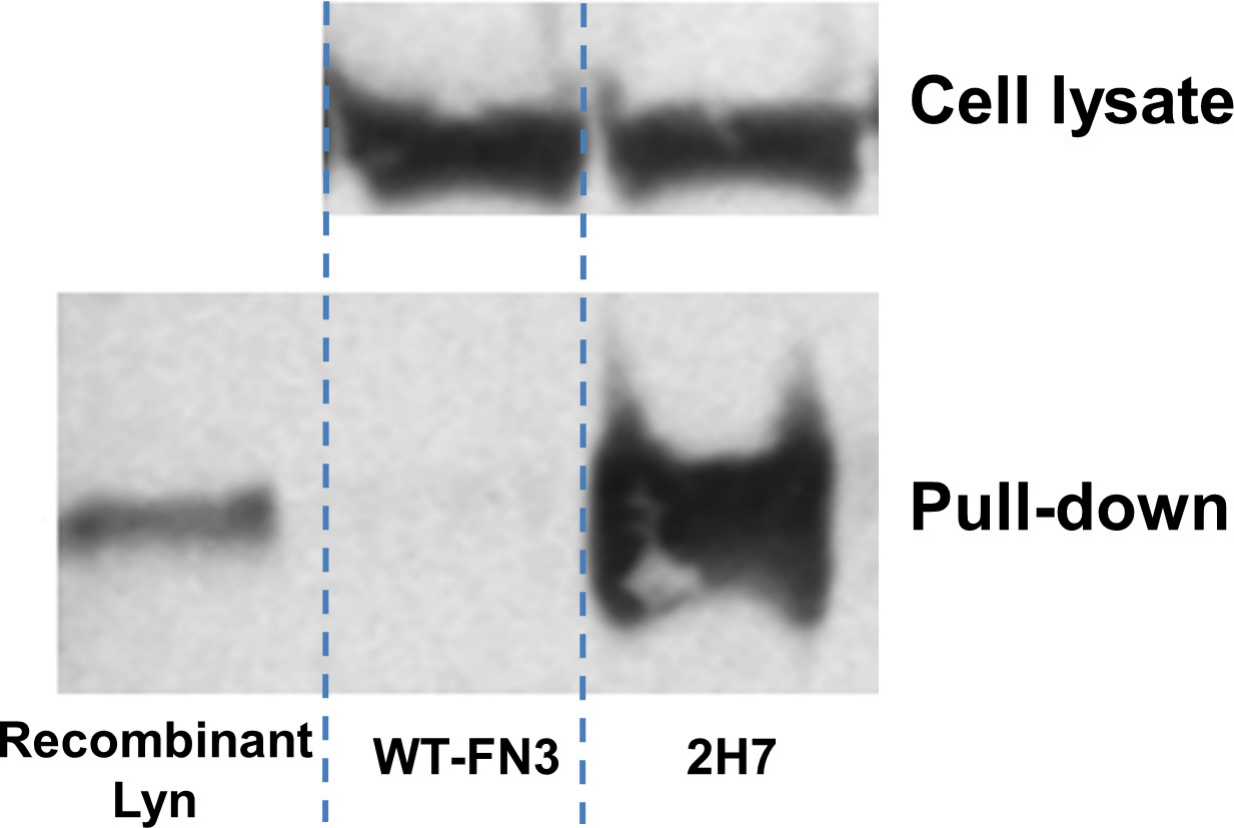
Pull-down of endogenous Lyn from human cells. Ramos cells were cultured and the clarified lysate was incubated with biotinylated 2H7 or wild-type FN3 (WT-FN3) monobody. Protein complex was then captured with streptavidin-coated magnetic beads and analyzed by Western blot. The Western blot included a recombinant Lyn protein as a positive control, along with the input and output samples of the pull-down experiment. The Lyn proteins were detected on the blot with a commercial anti-Lyn antibody.

To test if 2H7 was able to bind endogenous Lyn kinase in human mammalian cells, Burkitt's lymphoma cells (Ramos cells) [61] were cultured and the clarified lysate was incubated with biotinylated form of the 2H7 or wild-type FN3 (WT-FN3) monobody. Protein complex was then pulled down with streptavidin-coated magnetic beads and analyzed by Western blot. A recombinant full-length Lyn was included as a positive Western-blot control and the Lyn proteins were detected on the blot with a commercial anti-Lyn antibody. Compared to the WT-FN3, 2H7 was able to pull down the Lyn protein (Fig 8), demonstrating its potential utility, in combination with LC-MS/MS [62], for identifying proteins that interact with Lyn in mammalian cells.

**Fig 9:**
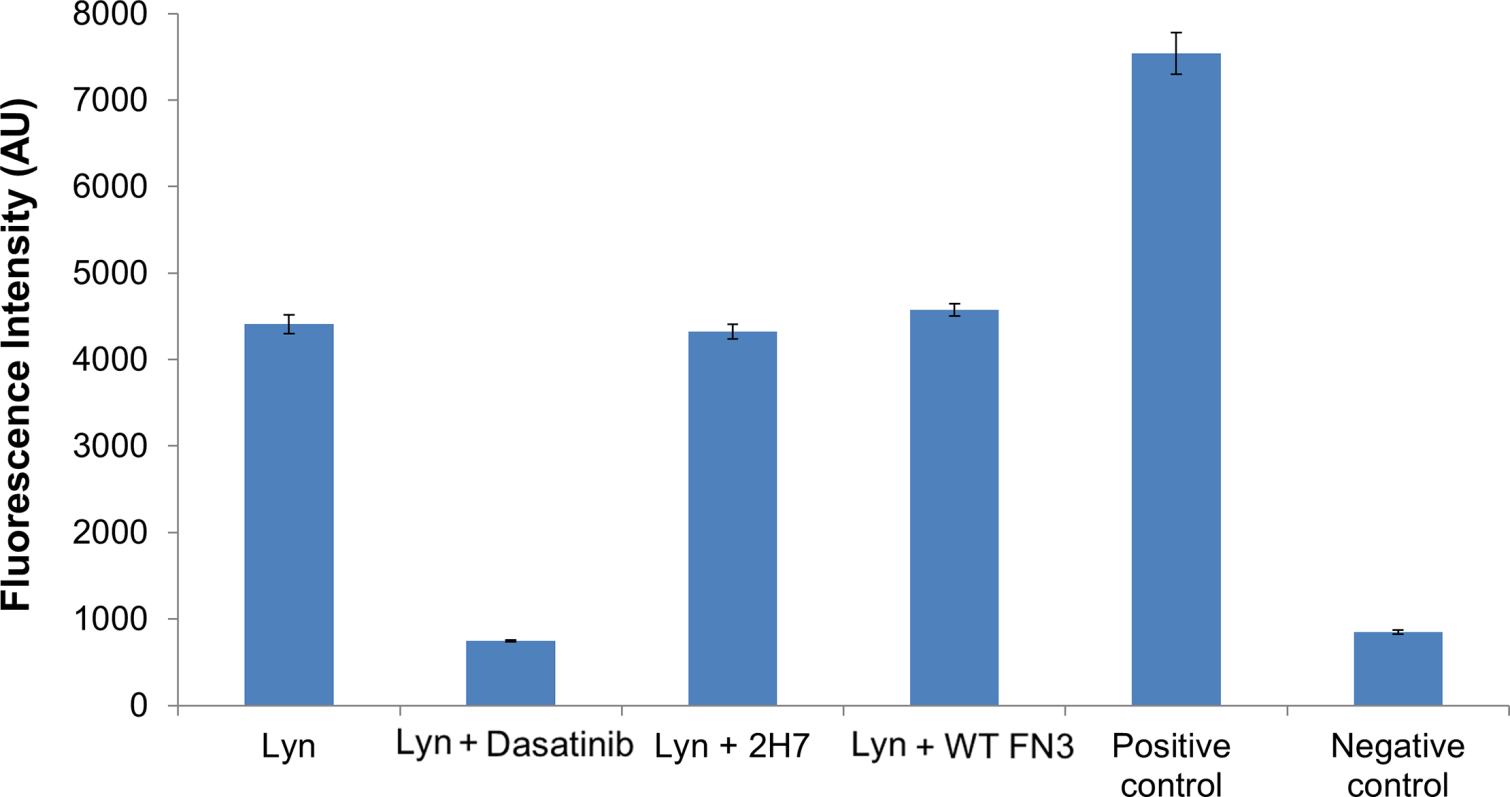
The 2H7 monobody did not perturb the kinase activity of Lyn. The kinase assay was performed with a Beacon Tyrosine kinase assay kit (Invitrogen), according to the manufacturer’s instructions. The assay with wild-type FN3 (Lyn + WT-FN3) was a non-perturbed control and the assay with Dasatinib (Lyn + Dasatinib) was a kinase-inhibited control. The assay with the Lyn kinase protein without any inhibitor or monobodies present served as the non-treatment control. Two internal controls of the assay were also included: when an anti-phosphopeptide antibody was excluded in the assay, a maximum fluorescence signal was observed (i.e., positive control), and when the recombinant Lyn protein was excluded in the assay, the lowest fluorescence signal was observed (i.e., negative control). Error bars are the standard deviations of triplicate measurements.

For using 2H7 monobody to study Lyn kinase, it was also desirable to determine if 2H7 interfered with its kinase activity. As shown in Fig 9, compared to the wild-type FN3 (WT-FN3) and the non-treated Lyn kinase, 2H7 did not reduce or increase the kinase activity of the Lyn. In contrast, Dasatinib, a drug that is used for treating chronic myelogenous leukemia and has a half maximal inhibitory concentration (IC_50_) of 8.5 nM against Lyn [63], completely inhibited Lyn kinase activity at the concentration of 10 μM.

## Discussion

Due to the limitations associated with conventional antibodies [64,65], there is a growing demand for recombinant affinity reagents with high specificity and affinity. *In vitro* display technologies, such as phage display, allow engineering such reagents for desired specificity and affinity to fulfill various applications. In this study, a recombinant FN3 monobody of high specificity and affinity was generated by affinity maturation with phage display. Through one round of mutagenesis, the affinity of the TA8 monobody was increased by more than 130-fold from 5.5 μM to 40–42 nM, but without losing its specificity for the Lyn SH3 domain.

Our success of dramatically improving the affinity of TA8, while preserving its specificity, can be possibly credited to the following reasons. First, its secondary library is large (1.2 x 10^9^) in size and covers most, if not all, of the possible combinations of NNK codons at the 6 randomized positions (32^6^ = 1.07 × 10^9^). Second, inclusion of the SH3 domains of Hck and Btk in the affinity selection may have removed cross-reactive variants. Third, the secondary library was constructed by randomizing only the residues of variable loops that were identified as non-crucial for binding. We reasoned that as the original TA8 clone had already exhibited remarkable specificity, mutations to crucial binding residues might dramatically alter the specificity. Fourth, besides the two non-crucial residues, the mutations of four residues flanking the original variable loops (i.e., Ser to Gly mutation in the FG loop), may have created extra contact sites that have contributed to the affinity increase.

In contrast to other proline-rich peptides isolated by phage display [66,67,68], TA8 monobody achieved a remarkable specificity towards the Lyn SH3 domain and understanding its mechanism may aid future efforts for engineering specific ligands to SH3 domains. Many natural ligands to SH3 domains achieve high specificities by interacting with a region outside of the canonical proline-binding pockets [60], and TA8 monobody may have achieved its specificity through a similar mechanism. This hypothesis is supported by the following evidence. First, the competition ELBA suggests that TA8 binds to a region outside of the proline-binding pockets on the Lyn SH3 domain. Second, this non-canonical region of the Lyn SH3 domain contains a Histidine residue (H30; Red squared in Fig 1C and highlighted in cyan Fig 7A) that is unique to Lyn among the 8 SFKs and interaction with this residue contributed to the higher specificity of Tip peptide towards the Lyn SH3 domain among SKFs [59]. Third, in another study, by targeting this unique histidine residue of Lyn SH3 domain, a synthetic peptide ligand was engineered to specifically recognize the Lyn SH3 domain over other SH3 domains [69].

In a previous publication [36], a monobody has been reported for recognizing the activated form of Src kinase by binding to its SH3 domain. In contrast to this finding, 2H7 did not exhibit any preferential binding to Lyn when it was activated (data not shown) by hydrogen peroxide [70]. Even more surprisingly, the monobody displayed preferential binding to the Lyn kinase when it was inhibited by Dasatinib (S2 Fig) or when it was inactivated by heat treatment (S3 Fig). The above observations are consistent with the two findings regard to the role of SH3 domain in regulating kinase activities of Src and Lyn. In one study [71], removal of the SH3 domain increases Src kinase activity, suggesting the intramolecular interaction between SH3 domain and other portions of the Src protein reduces its kinase activity [72,73]. In contrast, in the second study [74], removal of Lyn SH3 domain decreases its kinase activity, suggesting that the Lyn SH3 domain may act to enhance or maintain the kinase activity of Lyn. Taken together, these findings and our observations suggest that although Lyn and Src kinases have highly similar structures, their SH3 domains may play opposite roles in regulating their kinase activities.

As monobodies can be expressed in mammalian cells [37,75], the 2H7 monobody can be potentially used for many biological assays. For example, it can be potentially expressed in cells to pull down endogenous Lyn and identify interacting proteins through mass spectrometry, as what was done in a similar study [34]. This type of assay will be especially useful for identifying proteins that interact with Lyn through the unique domain [76] or SH2 domain [77,78]. In addition, although 2H7 did not perturb the Lyn kinase activity (Fig 9), it can still be used to disrupt the interactions between the SH3 domain with other cellular proteins, as shown by two previous studies [37,66].

An affinity reagent has to possess sufficient affinity and specificity to be useful in a biological assay. While there are many reported studies of improving affinities and specificities of the antibodies or other affinity reagents, a majority of those studies focus on either engineering affinities [79,80,81,82,83] or specificities of the reagents [84,85]. There are only a handful of studies that address both affinities and specificities of the reagents simultaneously [86,87]. In this study, we have utilized affinity selection of primary and secondary libraries to generate a monobody that binds to the Lyn SH3 domain with excellent affinity and specificity. This approach can be applicable for engineering the affinities and specificities of other affinity reagents, including antibodies or other alternative scaffolds.

## Supporting Information

S1 Fig Probing 150 human SH3 domains with a pan-specific monobody, 1F11. To demonstrate that many of the SH3 domains spotted on the array were functional, the arrays of 150 human SH3 domains (spotted in duplicate) were probed with 1F11. The names of the SFKs SH3 domains that bound the 1F11 monobody are labeled. The probing experiment was performed once.
(TIF)

S2 Fig The 2H7 monobody preferentially bound to the drug-inhibited Lyn. Protein of 2H7 monobody was immobilized on the microtiter plate, followed by blocking of the non-specific sites and addition of a recombinant GST-Lyn protein, with or without 10 μM Dasatinib, a Federal Drug Administration-approved inhibitor of BCR-ABL for treating chronic myelogenous leukemia [88]. The Dasatinib also inhibits Lyn kinase with an IC_50_ value of 8.5 nM [63]. An anti-GST antibody conjugated to horseradish peroxidase (Anti-GST HRP) was used to detect the GST tag. GST protein itself served as a negative control. The error bars are the standard deviations of triplicate measurements.
(TIF)

S3 Fig The 2H7 monobody preferentially bound to the heat denatured Lyn kinase. The 2H7 FN3 monobody was immobilized on the microtiter plate. A recombinant GST-Lyn kinase was diluted in PBS and heated at 95°C for 5 min. Then the denatured and non-treated GST-Lyn kinases were added into the blocked wells of microtiter plate, followed by detection with an anti-GST antibody conjugated to horseradish peroxidase (Anti-GST HRP). Casein was the blocking reagent and added into the plate for measuring the background binding. The error bars are the standard deviations of triplicate measurements.
(TIF)

S1 Text ELISA for testing the binding of 2H7 to inhibited or denatured Lyn kinase. (DOCX)

## References

[1] Parsons SJ, Parsons JT (2004) Src family kinases, key regulators of signal transduction. Oncogene 23: 7906–7909. 15489908 10.1038/sj.onc.1208160

[2] Thomas SM, Brugge JS (1997) Cellular functions regulated by Src family kinases. Annu Rev Cell Dev Biol 13: 513–609. 9442882 10.1146/annurev.cellbio.13.1.513

[3] Liu BA, Shah E, Jablonowski K, Stergachis A, Engelmann B, Nash PD (2011) The SH2 domain-containing proteins in 21 species establish the provenance and scope of phosphotyrosine signaling in eukaryotes. Sci Signal 4: ra83 10.1126/scisignal.2002105 22155787 PMC4255630

[4] Manning G, Whyte DB, Martinez R, Hunter T, Sudarsanam S (2002) The protein kinase complement of the human genome. Science 298: 1912–1934. 12471243 10.1126/science.1075762

[5] Bolen JB, Brugge JS (1997) Leukocyte protein tyrosine kinases: potential targets for drug discovery. Annu Rev Immunol 15: 371–404. 9143693 10.1146/annurev.immunol.15.1.371

[6] Xiao W, Nishimoto H, Hong H, Kitaura J, Nunomura S, Maeda-Yamamoto M, et al (2005) Positive and negative regulation of mast cell activation by Lyn via the FcepsilonRI. J Immunol 175: 6885–6892. 16272347 10.4049/jimmunol.175.10.6885 PMC1415265

[7] Barua D, Hlavacek WS, Lipniacki T (2012) A computational model for early events in B cell antigen receptor signaling: analysis of the roles of Lyn and Fyn. J Immunol 189: 646–658. 10.4049/jimmunol.1102003 22711887 PMC3392547

[8] Katagiri K, Yokoyama KK, Yamamoto T, Omura S, Irie S, Katagiri T (1996) Lyn and Fgr protein-tyrosine kinases prevent apoptosis during retinoic acid-induced granulocytic differentiation of HL-60 cells. J Biol Chem 271: 11557–11562. 8626717 10.1074/jbc.271.19.11557

[9] Yoo SK, Freisinger CM, Lebert DC, Huttenlocher A (2012) Early redox, Src family kinase, and calcium signaling integrate wound responses and tissue regeneration in zebrafish. J Cell Biol 199: 225–234. 10.1083/jcb.201203154 23045550 PMC3471241

[10] Wang YH, Fan L, Wang L, Zhang R, Zou ZJ, Fang C, et al (2012) The expression levels of Lyn, Syk, PLCgamma2 and ERK in patients with chronic lymphocytic leukemia, and higher levels of Lyn associate with a shorter treatment free survival. Leuk Lymphoma. 10.3109/10428194.2012.736983 23039362

[11] Ingley E (2012) Functions of the Lyn tyrosine kinase in health and disease. Cell Commun Signal 10: 21 10.1186/1478-811X-10-21 22805580 PMC3464935

[12] Tabe Y, Jin L, Iwabuchi K, Wang RY, Ichikawa N, Miida T, et al (2012) Role of stromal microenvironment in nonpharmacological resistance of CML to imatinib through Lyn/CXCR4 interactions in lipid rafts. Leukemia 26: 883–892. 10.1038/leu.2011.291 22005789

[13] Tsantikos E, Maxwell MJ, Kountouri N, Harder KW, Tarlinton DM, Hibbs ML (2012) Genetic interdependence of Lyn and negative regulators of B cell receptor signaling in autoimmune disease development. J Immunol 189: 1726–1736. 10.4049/jimmunol.1103427 22798664

[14] Huston JS, Levinson D, Mudgett-Hunter M, Tai MS, Novotny J, Margolies MN, et al (1988) Protein engineering of antibody binding sites: recovery of specific activity in an anti-digoxin single-chain Fv analogue produced in Escherichia coli. Proc Natl Acad Sci U S A 85: 5879–5883. 3045807 10.1073/pnas.85.16.5879 PMC281868

[15] Miersch S, Sidhu SS (2012) Synthetic antibodies: concepts, potential and practical considerations. Methods 57: 486–498. 10.1016/j.ymeth.2012.06.012 22750306

[16] de Marco A (2011) Biotechnological applications of recombinant single-domain antibody fragments. Microb Cell Fact 10: 44 10.1186/1475-2859-10-44 21658216 PMC3123181

[17] Nilsson FY, Tolmachev V (2007) Affibody molecules: new protein domains for molecular imaging and targeted tumor therapy. Curr Opin Drug Discov Devel 10: 167–175. 17436552

[18] Skerra A (2008) Alternative binding proteins: anticalins—harnessing the structural plasticity of the lipocalin ligand pocket to engineer novel binding activities. FEBS J 275: 2677–2683. 10.1111/j.1742-4658.2008.06439.x 18435758

[19] Boersma YL, Pluckthun A (2011) DARPins and other repeat protein scaffolds: advances in engineering and applications. Curr Opin Biotechnol 22: 849–857. 10.1016/j.copbio.2011.06.004 21715155

[20] Koide A, Bailey CW, Huang X, Koide S (1998) The fibronectin type III domain as a scaffold for novel binding proteins. J Mol Biol 284: 1141–1151. 9837732 10.1006/jmbi.1998.2238

[21] Lofblom J, Feldwisch J, Tolmachev V, Carlsson J, Stahl S, Frejd FY (2010) Affibody molecules: engineered proteins for therapeutic, diagnostic and biotechnological applications. FEBS Lett 584: 2670–2680. 10.1016/j.febslet.2010.04.014 20388508

[22] Lu Z, Murray KS, Van Cleave V, LaVallie ER, Stahl ML, McCoy JM (1995) Expression of thioredoxin random peptide libraries on the Escherichia coli cell surface as functional fusions to flagellin: a system designed for exploring protein-protein interactions. Biotechnology (N Y) 13: 366–372. 9634778 10.1038/nbt0495-366

[23] Smith GP (1985) Filamentous fusion phage: novel expression vectors that display cloned antigens on the virion surface. Science 228: 1315–1317. 4001944 10.1126/science.4001944

[24] Roberts RW, Szostak JW (1997) RNA-peptide fusions for the in vitro selection of peptides and proteins. Proc Natl Acad Sci U S A 94: 12297–12302. 9356443 10.1073/pnas.94.23.12297 PMC24913

[25] Mattheakis LC, Bhatt RR, Dower WJ (1994) An in vitro polysome display system for identifying ligands from very large peptide libraries. Proc Natl Acad Sci U S A 91: 9022–9026. 7522328 10.1073/pnas.91.19.9022 PMC44739

[26] Boder ET, Wittrup KD (1997) Yeast surface display for screening combinatorial polypeptide libraries. Nat Biotechnol 15: 553–557. 9181578 10.1038/nbt0697-553

[27] Bradbury A, Pluckthun A (2015) Reproducibility: Standardize antibodies used in research. Nature 518: 27–29. 10.1038/518027a 25652980

[28] Gilbreth RN, Truong K, Madu I, Koide A, Wojcik JB, Li NS, et al (2011) Isoform-specific monobody inhibitors of small ubiquitin-related modifiers engineered using structure-guided library design. Proc Natl Acad Sci U S A 108: 7751–7756. 10.1073/pnas.1102294108 21518904 PMC3093456

[29] Koide A, Abbatiello S, Rothgery L, Koide S (2002) Probing protein conformational changes in living cells by using designer binding proteins: application to the estrogen receptor. Proc Natl Acad Sci U S A 99: 1253–1258. 11818562 10.1073/pnas.032665299 PMC122176

[30] Hackel BJ, Kapila A, Wittrup KD (2008) Picomolar affinity fibronectin domains engineered utilizing loop length diversity, recursive mutagenesis, and loop shuffling. J Mol Biol 381: 1238–1252. 10.1016/j.jmb.2008.06.051 18602401 PMC2840393

[31] Karatan E, Merguerian M, Han Z, Scholle MD, Koide S, Kay BK (2004) Molecular recognition properties of FN3 monobodies that bind the Src SH3 domain. Chem Biol 11: 835–844. 15217616 10.1016/j.chembiol.2004.04.009

[32] Koide A, Gilbreth RN, Esaki K, Tereshko V, Koide S (2007) High-affinity single-domain binding proteins with a binary-code interface. Proc Natl Acad Sci U S A 104: 6632–6637. 17420456 10.1073/pnas.0700149104 PMC1871837

[33] Olson CA, Liao HI, Sun R, Roberts RW (2008) mRNA display selection of a high-affinity, modification-specific phospho-IkappaBalpha-binding fibronectin. ACS Chem Biol 3: 480–485. 10.1021/cb800069c 18590330 PMC2962918

[34] Wojcik J, Hantschel O, Grebien F, Kaupe I, Bennett KL, Barkinge J, et al (2010) A potent and highly specific FN3 monobody inhibitor of the Abl SH2 domain. Nat Struct Mol Biol 17: 519–527. 10.1038/nsmb.1793 20357770 PMC2926940

[35] Park SH, Park S, Kim DY, Pyo A, Kimura RH, Sathirachinda A, et al (2015) Isolation and Characterization of a Monobody with a Fibronectin Domain III Scaffold That Specifically Binds EphA2. PLoS One 10: e0132976 10.1371/journal.pone.0132976 26177208 PMC4503726

[36] Gulyani A, Vitriol E, Allen R, Wu J, Gremyachinskiy D, Lewis S, et al (2011) A biosensor generated via high-throughput screening quantifies cell edge Src dynamics. Nat Chem Biol 7: 437–444. 10.1038/nchembio.585 21666688 PMC3135387

[37] Cochran JN, Diggs PV, Nebane NM, Rasmussen L, White EL, Bostwick R, et al (2014) AlphaScreen HTS and live-cell bioluminescence resonance energy transfer (BRET) assays for identification of Tau-Fyn SH3 interaction inhibitors for Alzheimer disease. J Biomol Screen 19: 1338–1349. 10.1177/1087057114547232 25156556 PMC4318572

[38] Kunkel TA (1985) Rapid and efficient site-specific mutagenesis without phenotypic selection. Proc Natl Acad Sci U S A 82: 488–492. 3881765 10.1073/pnas.82.2.488 PMC397064

[39] Huang R, Fang P, Kay BK (2012) Improvements to the Kunkel mutagenesis protocol for constructing primary and secondary phage-display libraries. Methods. 10.1016/j.ymeth.2012.08.008 PMC3491174 22959950

[40] Pershad K, Sullivan MA, Kay BK (2011) Drop-out phagemid vector for switching from phage displayed affinity reagents to expression formats. Anal Biochem 412: 210–216. 10.1016/j.ab.2011.02.006 21315061 PMC3068214

[41] Schlesinger MJ, Andersen L (1968) Multiple molecular forms of the alkaline phosphatase of Escherichia coli. Ann N Y Acad Sci 151: 159–170. 4887360 10.1111/j.1749-6632.1968.tb11886.x

[42] Arbabi Ghahroudi M, Desmyter A, Wyns L, Hamers R, Muyldermans S (1997) Selection and identification of single domain antibody fragments from camel heavy-chain antibodies. FEBS Lett 414: 521–526. 9323027 10.1016/s0014-5793(97)01062-4

[43] Huang R, Fang P, Kay BK (2012) Isolation of monobodies that bind specifically to the SH3 domain of the Fyn tyrosine protein kinase. N Biotechnol 29: 526–533. 10.1016/j.nbt.2011.11.015 22155429 PMC3319870

[44] Smith DB, Johnson KS (1988) Single-step purification of polypeptides expressed in Escherichia coli as fusions with glutathione S-transferase. Gene 67: 31–40. 3047011 10.1016/0378-1119(88)90005-4

[45] Kay BK, Thai S, Volgina VV (2009) High-throughput biotinylation of proteins. Methods Mol Biol 498: 185–196. 10.1007/978-1-59745-196-3_13 18988027 PMC3223083

[46] Bauer F, Schweimer K, Meiselbach H, Hoffmann S, Rösch P, Sticht H (2005) Structural characterization of Lyn-SH3 domain in complex with a herpesviral protein reveals an extended recognition motif that enhances binding affinity. Protein Science 14: 2487–2498. 16155203 10.1110/ps.051563605 PMC2253286

[47] Bass SH, Mulkerrin MG, Wells JA (1991) A systematic mutational analysis of hormone-binding determinants in the human growth hormone receptor. Proc Natl Acad Sci U S A 88: 4498–4502. 2034689 10.1073/pnas.88.10.4498 PMC51688

[48] Bradford MM (1976) A rapid and sensitive method for the quantitation of microgram quantities of protein utilizing the principle of protein-dye binding. Anal Biochem 72: 248–254. 942051 10.1016/0003-2697(76)90527-3

[49] Viguera AR, Arrondo JL, Musacchio A, Saraste M, Serrano L (1994) Characterization of the interaction of natural proline-rich peptides with five different SH3 domains. Biochemistry 33: 10925–10933. 8086409 10.1021/bi00202a011

[50] Rickles RJ, Botfield MC, Zhou XM, Henry PA, Brugge JS, Zoller MJ (1995) Phage display selection of ligand residues important for Src homology 3 domain binding specificity. Proc Natl Acad Sci U S A 92: 10909–10913. 7479908 10.1073/pnas.92.24.10909 PMC40540

[51] Sparks AB, Rider JE, Hoffman NG, Fowlkes DM, Quillam LA, Kay BK (1996) Distinct ligand preferences of Src homology 3 domains from Src, Yes, Abl, Cortactin, p53bp2, PLCgamma, Crk, and Grb2. Proc Natl Acad Sci U S A 93: 1540–1544. 8643668 10.1073/pnas.93.4.1540 PMC39976

[52] Karkkainen S, Hiipakka M, Wang JH, Kleino I, Vaha-Jaakkola M, Renkema GH, et al (2006) Identification of preferred protein interactions by phage-display of the human Src homology-3 proteome. EMBO Rep 7: 186–191. 16374509 10.1038/sj.embor.7400596 PMC1369250

[53] Colwill K, Graslund S (2011) A roadmap to generate renewable protein binders to the human proteome. Nat Methods 8: 551–558. 10.1038/nmeth.1607 21572409

[54] Pierce MM, Raman CS, Nall BT (1999) Isothermal titration calorimetry of protein-protein interactions. Methods 19: 213–221. 10527727 10.1006/meth.1999.0852

[55] Sparks AB, Hoffman NG, McConnell SJ, Fowlkes DM, Kay BK (1996) Cloning of ligand targets: systematic isolation of SH3 domain-containing proteins. Nat Biotechnol 14: 741–744. 9630982 10.1038/nbt0696-741

[56] Mayer BJ (2001) SH3 domains: complexity in moderation. J Cell Sci 114: 1253–1263. 11256992 10.1242/jcs.114.7.1253

[57] Feng S, Chen JK, Yu H, Simon JA, Schreiber SL (1994) Two binding orientations for peptides to the Src SH3 domain: development of a general model for SH3-ligand interactions. Science 266: 1241–1247. 7526465 10.1126/science.7526465

[58] Yu H, Chen JK, Feng S, Dalgarno DC, Brauer AW, Schreiber SL (1994) Structural basis for the binding of proline-rich peptides to SH3 domains. Cell 76: 933–945. 7510218 10.1016/0092-8674(94)90367-0

[59] Schweimer K, Hoffmann S, Bauer F, Friedrich U, Kardinal C, Feller SM, et al (2002) Structural Investigation of the Binding of a Herpesviral Protein to the SH3 Domain of Tyrosine Kinase Lck†,‡. Biochemistry 41: 5120–5130. 11955060 10.1021/bi015986j

[60] Saksela K, Permi P (2012) SH3 domain ligand binding: What's the consensus and where's the specificity? FEBS Lett 586: 2609–2614. 10.1016/j.febslet.2012.04.042 22710157

[61] Molyneux EM, Rochford R, Griffin B, Newton R, Jackson G, Menon G, et al (2012) Burkitt's lymphoma. Lancet 379: 1234–1244. 10.1016/S0140-6736(11)61177-X 22333947

[62] Pitt JJ (2009) Principles and applications of liquid chromatography-mass spectrometry in clinical biochemistry. Clin Biochem Rev 30: 19–34. 19224008 PMC2643089

[63] Nam S, Kim D, Cheng JQ, Zhang S, Lee JH, Buettner R, et al (2005) Action of the Src family kinase inhibitor, dasatinib (BMS-354825), on human prostate cancer cells. Cancer Res 65: 9185–9189. 16230377 10.1158/0008-5472.CAN-05-1731

[64] Jensen BC, Swigart PM, Simpson PC (2009) Ten commercial antibodies for alpha-1-adrenergic receptor subtypes are nonspecific. Naunyn Schmiedebergs Arch Pharmacol 379: 409–412. 10.1007/s00210-008-0368-6 18989658 PMC2653258

[65] Couchman JR (2009) Commercial antibodies: the good, bad, and really ugly. J Histochem Cytochem 57: 7–8. 10.1369/jhc.2008.952820 18854593 PMC2605718

[66] Stauffer TP, Martenson CH, Rider JE, Kay BK, Meyer T (1997) Inhibition of Lyn function in mast cell activation by SH3 domain binding peptides. Biochemistry 36: 9388–9394. 9235982 10.1021/bi970781p

[67] Feng S, Kasahara C, Rickles RJ, Schreiber SL (1995) Specific interactions outside the proline-rich core of two classes of Src homology 3 ligands. Proc Natl Acad Sci U S A 92: 12408–12415. 8618911 10.1073/pnas.92.26.12408 PMC40367

[68] Rickles RJ, Botfield MC, Weng Z, Taylor JA, Green OM, Brugge JS, et al (1994) Identification of Src, Fyn, Lyn, PI3K and Abl SH3 domain ligands using phage display libraries. EMBO J 13: 5598–5604. 7988556 10.1002/j.1460-2075.1994.tb06897.x PMC395523

[69] Vohidov F, Knudsen SE, Leonard PG, Ohata J, Wheadon MJ, Popp BV, et al (2015) Potent and selective inhibition of SH3 domains with dirhodium metalloinhibitors. Chem Sci 6: 4778–4783. 29142714 10.1039/c5sc01602a PMC5667506

[70] Yoo SK, Starnes TW, Deng Q, Huttenlocher A (2011) Lyn is a redox sensor that mediates leukocyte wound attraction in vivo. Nature 480: 109–112. 10.1038/nature10632 22101434 PMC3228893

[71] Seidel-Dugan C, Meyer BE, Thomas SM, Brugge JS (1992) Effects of SH2 and SH3 deletions on the functional activities of wild-type and transforming variants of c-Src. Mol Cell Biol 12: 1835–1845. 1549129 10.1128/mcb.12.4.1835-1845.1992 PMC369627

[72] Young MA, Gonfloni S, Superti-Furga G, Roux B, Kuriyan J (2001) Dynamic coupling between the SH2 and SH3 domains of c-Src and Hck underlies their inactivation by C-terminal tyrosine phosphorylation. Cell 105: 115–126. 11301007 10.1016/s0092-8674(01)00301-4

[73] Boggon TJ, Eck MJ (2004) Structure and regulation of Src family kinases. Oncogene 23: 7918–7927. 15489910 10.1038/sj.onc.1208081

[74] Abrams CS, Zhao W (1995) SH3 domains specifically regulate kinase activity of expressed Src family proteins. J Biol Chem 270: 333–339. 7529230 10.1074/jbc.270.1.333

[75] Grebien F, Hantschel O, Wojcik J, Kaupe I, Kovacic B, Wyrzucki AM, et al (2011) Targeting the SH2-kinase interface in Bcr-Abl inhibits leukemogenesis. Cell 147: 306–319. 10.1016/j.cell.2011.08.046 22000011 PMC3202669

[76] Adachi T, Pazdrak K, Stafford S, Alam R (1999) The mapping of the Lyn kinase binding site of the common beta subunit of IL-3/granulocyte-macrophage colony-stimulating factor/IL-5 receptor. J Immunol 162: 1496–1501. 9973406

[77] Malek SN, Desiderio S (1993) SH2 domains of the protein-tyrosine kinases Blk, Lyn, and Fyn(T) bind distinct sets of phosphoproteins from B lymphocytes. J Biol Chem 268: 22557–22565. 8226767

[78] Jin LL, Wybenga-Groot LE, Tong J, Taylor P, Minden MD, Trudel S, et al (2015) Tyrosine phosphorylation of the Lyn Src homology 2 (SH2) domain modulates its binding affinity and specificity. Mol Cell Proteomics 14: 695–706. 10.1074/mcp.M114.044404 25587033 PMC4349988

[79] Gram H, Marconi LA, Barbas CF III, Collet TA, Lerner RA, Kang AS (1992) In vitro selection and affinity maturation of antibodies from a naive combinatorial immunoglobulin library. Proc Natl Acad Sci U S A 89: 3576–3580. 1565653 10.1073/pnas.89.8.3576 PMC48911

[80] Marks JD, Griffiths AD, Malmqvist M, Clackson TP, Bye JM, Winter G (1992) By-passing immunization: building high affinity human antibodies by chain shuffling. Biotechnology (N Y) 10: 779–783. 1368267 10.1038/nbt0792-779

[81] Hawkins RE, Russell SJ, Winter G (1992) Selection of phage antibodies by binding affinity. Mimicking affinity maturation. J Mol Biol 226: 889–896. 1507232 10.1016/0022-2836(92)90639-2

[82] Douthwaite JA, Sridharan S, Huntington C, Hammersley J, Marwood R, Hakulinen JK, et al (2015) Affinity maturation of a novel antagonistic human monoclonal antibody with a long VH CDR3 targeting the Class A GPCR formyl-peptide receptor 1. MAbs 7: 152–166. 10.4161/19420862.2014.985158 25484051 PMC4622665

[83] Imai S, Naito S, Takahashi T, Yamauchi A, Nakamura E, Sato M, et al (2015) Development of an ultrasensitive immunoassay using affinity maturated antibodies for the measurement of rodent insulin. Anal Biochem 473: 72–79. 10.1016/j.ab.2014.12.003 25524616

[84] Fagete S, Ravn U, Gueneau F, Magistrelli G, Kosco-Vilbois MH, Fischer N (2009) Specificity tuning of antibody fragments to neutralize two human chemokines with a single agent. MAbs 1: 288–296. 20069756 10.4161/mabs.1.3.8527 PMC2726596

[85] Bostrom J, Yu SF, Kan D, Appleton BA, Lee CV, Billeci K, et al (2009) Variants of the antibody herceptin that interact with HER2 and VEGF at the antigen binding site. Science 323: 1610–1614. 10.1126/science.1165480 19299620

[86] Roberts S, Cheetham JC, Rees AR (1987) Generation of an antibody with enhanced affinity and specificity for its antigen by protein engineering. Nature 328: 731–734. 3614380 10.1038/328731a0

[87] Chames P, Coulon S, Baty D (1998) Improving the affinity and the fine specificity of an anti-cortisol antibody by parsimonious mutagenesis and phage display. J Immunol 161: 5421–5429. 9820517

[88] Talpaz M, Shah NP, Kantarjian H, Donato N, Nicoll J, Paquette R, et al (2006) Dasatinib in imatinib-resistant Philadelphia chromosome-positive leukemias. N Engl J Med 354: 2531–2541. 16775234 10.1056/NEJMoa055229

