# Supplementary figures and images for: Directed Evolution of a Highly Specific FN3 Monobody to the SH3 Domain of Human Lyn Tyrosine Kinase
Source: PLoS One. 2016 Jan 5;11(1):e0145872. doi: 10.1371/journal.pone.0145872 (PMC4701441; doi:10.1371/journal.pone.0145872)

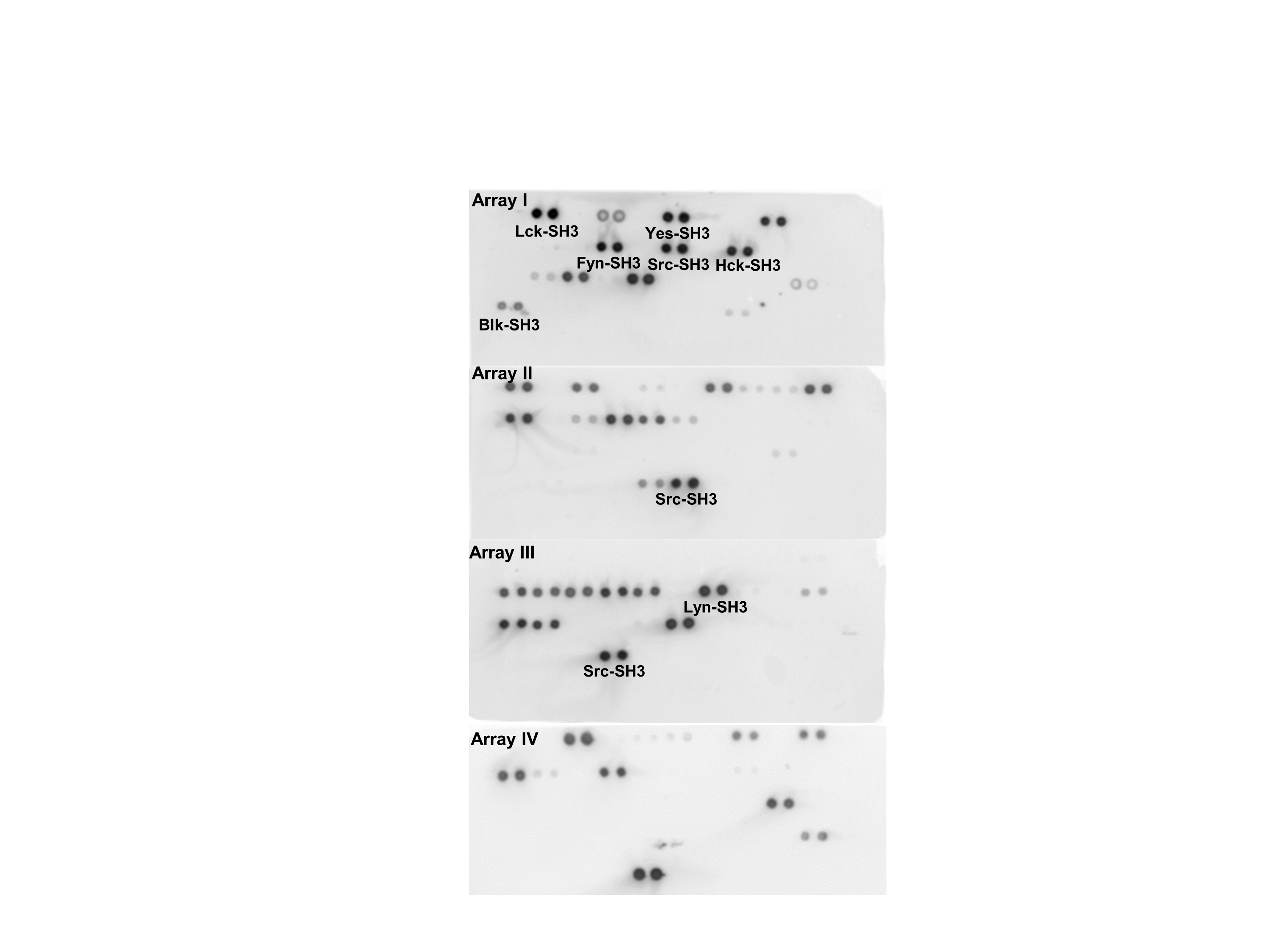

Supplement: S1 Fig — To demonstrate that many of the SH3 domains spotted on the array were functional, the arrays of 150 human SH3 domains (spotted in duplicate) were probed with 1F11. The names of the SFKs SH3 domains that bound the 1F11 monobody are labeled. The probing experiment was performed once. (TIF) [file pone.0145872.s001.TIF]

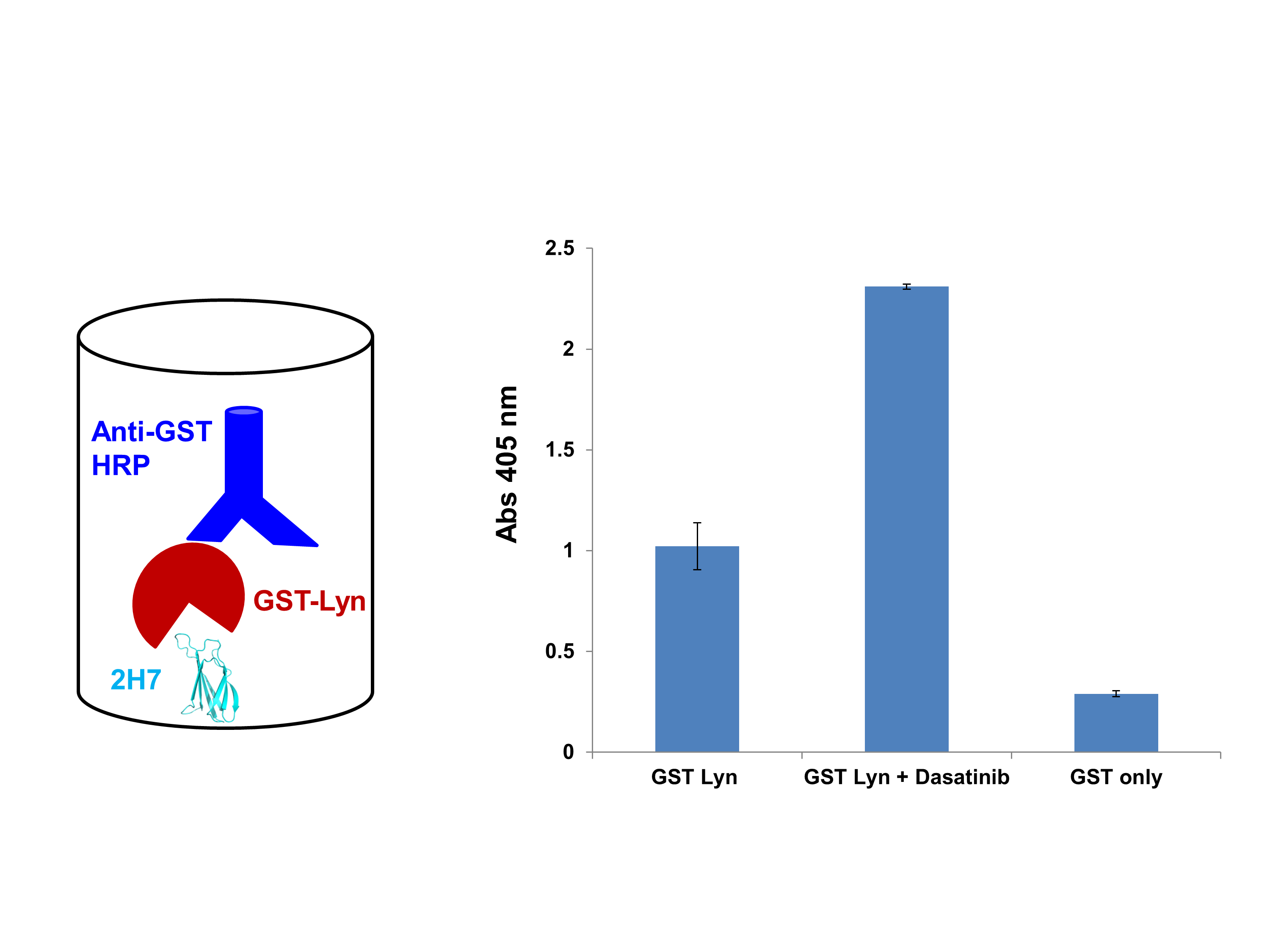

Supplement: S2 Fig — Protein of 2H7 monobody was immobilized on the microtiter plate, followed by blocking of the non-specific sites and addition of a recombinant GST-Lyn protein, with or without 10 μM Dasatinib, a Federal Drug Administration-approved inhibitor of BCR-ABL for treating chronic myelogenous leukemia [88]. The Dasatinib also inhibits Lyn kinase with an IC50 value of 8.5 nM [63]. An anti-GST antibody conjugated to horseradish peroxidase (Anti-GST HRP) was used to detect the GST tag. GST protein itself served as a negative control. The error bars are the standard deviations of triplicate measurements. (TIF) [file pone.0145872.s002.TIF]

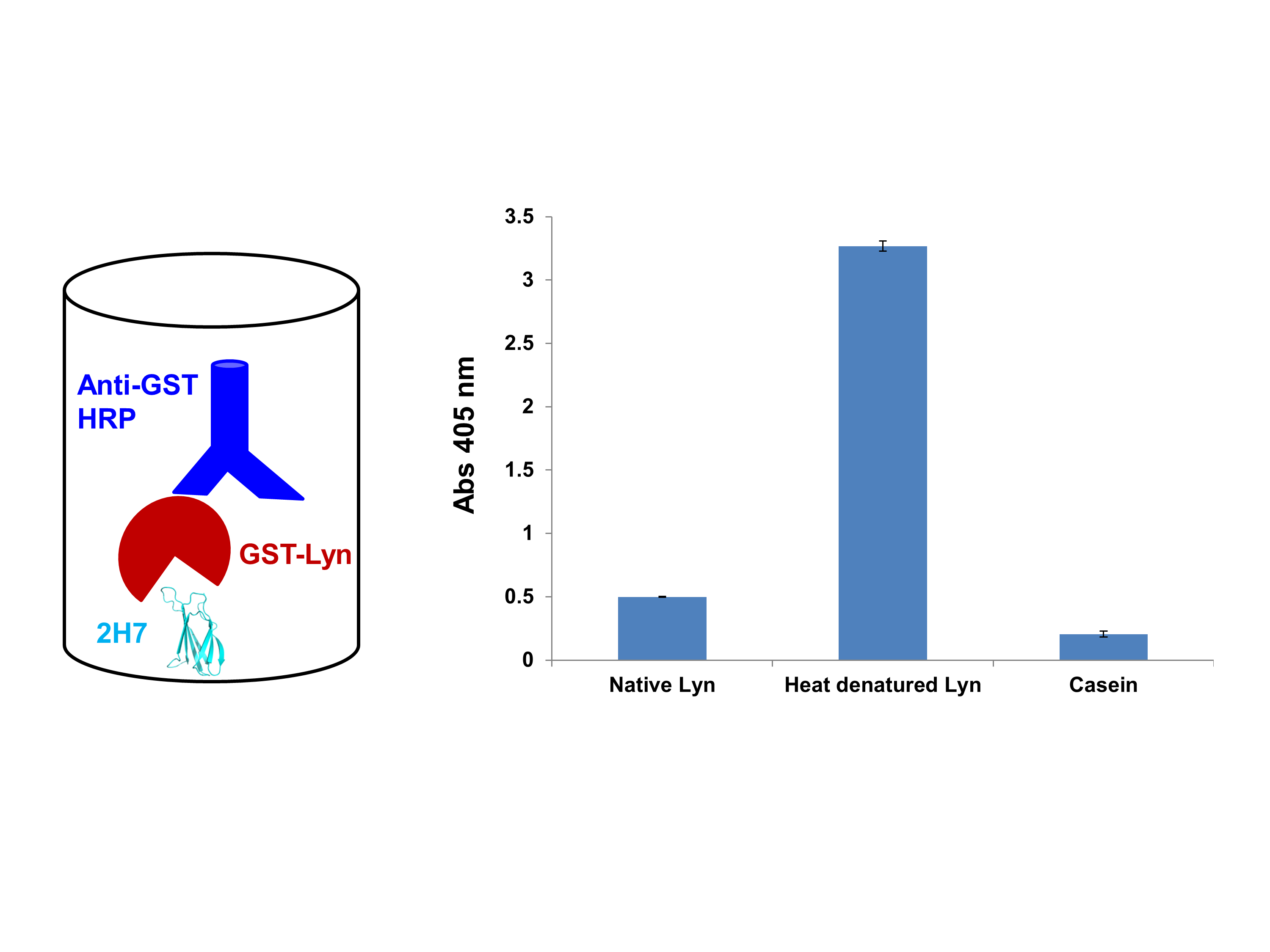

Supplement: S3 Fig — The 2H7 FN3 monobody was immobilized on the microtiter plate. A recombinant GST-Lyn kinase was diluted in PBS and heated at 95°C for 5 min. Then the denatured and non-treated GST-Lyn kinases were added into the blocked wells of microtiter plate, followed by detection with an anti-GST antibody conjugated to horseradish peroxidase (Anti-GST HRP). Casein was the blocking reagent and added into the plate for measuring the background binding. The error bars are the standard deviations of triplicate measurements. (TIF) [file pone.0145872.s003.TIF]
