# Supplementary material for: Directed Evolution of a Highly Specific FN3 Monobody to the SH3 Domain of Human Lyn Tyrosine Kinase
Source: PLoS One. 2016 Jan 5;11(1):e0145872. doi: 10.1371/journal.pone.0145872 (PMC4701441; doi:10.1371/journal.pone.0145872)
Supplement: S1 Text — (DOCX) [file pone.0145872.s004.docx]

**Supporting Materials and Methods**

**ELISA for testing the binding of 2H7 to inhibited or denatured Lyn kinase**

Purified protein of 2H7 was diluted to 50 µg/mL in PBS and added to the wells of Nunc Maxisorp microtiter plate (Thermo Fisher Scientific; Waltham, MA, # 44240421) for overnight coating. The next day, the microtiter plate wells were blocked with casein (Thermo Fisher Scientific, # PI-37528) for two hours (h). During the plate blocking, GST-tagged Lyn kinase (Sigma-Aldrich; St. Louis, MO, # L2670) was diluted in PBS + 0.1% Tween 20 to a final concentration of 4 µg/mL. The diluted Lyn kinase was divided into two aliquots, one of which was supplemented with Dasatinib [1] (Selleck Chemicals; Houston, TX, # S1021), an inhibitor of Lyn kinase, at a final concentration of 10 µM. After 30 minutes (min) incubation at room temperature, the GST-Lyn kinase with or without Dasatinib were added to the microtiter plate wells for 1h incubation. As the negative control, GST tag protein itself was included in the assay. Anti-GST antibody conjugated to horseradish peroxidase (Anti-GST HRP; GE Healthcare life sciencies, # RPN1236) was added to the wells for 1h incubation, followed by three washes with PBS + 0.1% tween 20 and addition of 2,2'-azino-bis(3-ethylbenzothiazoline-6-sulphonic acid) (ABTS) and 0.3% hydrogen peroxide for detection. The resulting absorbance was determined at 405 nm with a microtiter plate spectrophotometer (BMG Labtech; Cary, NC).

For ELISA to determine the binding of 2H7 monobody to the heat denatured Lyn kinase, purified protein of 2H7 monobody was immobilized on the Nunc microtiter plate (Thermo Fisher Scientific) as described above. The next day, the microtiter plate was blocked with casein (Thermo Fisher Scientific) for 2h. In the meantime, GST-Lyn kinase (Sigma-Aldrich) was diluted in PBS to 3 μg/mL, divided into two aliquots and one aliquot was heat denatured at 95 °C for 5 min. Then both the denatured and non-treated GST-Lyn kinases were added to the microtiter plate wells for 1h incubation. The subsequent steps of ELISA were performed as described above for the binding assay to inhibited Lyn kinase.

**Reference**

1. Talpaz M, Shah NP, Kantarjian H, Donato N, Nicoll J, Paquette R, et al. (2006) Dasatinib in imatinib-resistant Philadelphia chromosome-positive leukemias. N Engl J Med 354: 2531-2541.
